# eNAMPT is a plasma biomarker of COVID-19 severity and ARDS progression linked to increased immune effector cell expression

**DOI:** 10.1371/journal.pone.0360244

**Published:** 2026-11-03

**Authors:** Jason Canizales Galaviz, Amir Khan, Bochra Tourki, Susannah Patarroyo-White, Nathan H. Johnson, Alejandro Arjona, Christian Bime, Kenneth S. Knox, Mehdi S. Mirsaeidi, Brenda Juan Guardela, Jose D. Herazo-Maya, Nancy G. Casanova, Joe G. N. Garcia

**Affiliations:** 1 Center for Inflammation Science and Systems Medicine, The Herbert Wertheim University of Florida Scripps Research Institute, Jupiter, Florida, United States of America; 2 University of Arizona College of Medicine, Phoenix, Arizona, United States of America; 3 Division of Pulmonary, Critical Care and Sleep Medicine, University of Texas at Austin, Austin, Texas. United States of America; 4 Division of Pulmonary, Critical Care and Sleep Medicine. Department of Internal Medicine, Morsani College of Medicine, University of South Florida, Ubben Center for Pulmonary Fibrosis Research, Tampa, Florida., United States of America; Ann and Robert H Lurie Children’s Hospital of Chicago, Northwestern University, UNITED STATES OF AMERICA

## Abstract

**Background:**

The COVID-19 pandemic highlighted the critical unmet need for novel targets, biomarkers, and effective pharmacotherapies to reduce the severity/mortality of COVID pneumonia and progression to acute respiratory distress syndrome (ARDS). Extracellular NAMPT (eNAMPT) is a druggable DAMP and TLR4 ligand centrally involved in ARDS pathobiology. We explored the utility of plasma eNAMPT measurements as a potential COVID-19 biomarker linked to ARDS progression and disease severity and investigated the potential cellular source for eNAMPT release/secretion in COVID-19 subjects.

**Methods:**

A plasma biomarker panel of inflammatory cytokines (IL-1β, IL-6, IL-8, IL-1RA, TNF-α), DAMPs (eNAMPT, MIF) and vascular proteins (ANG-2, PSGL-1) were evaluated in three distinct COVID-19-positive cohorts (Miami, Arizona, Tampa) (n = 277) and healthy control subjects (n = 214). Single-cell RNA sequencing (scRNA-seq) publicly available on peripheral blood mononuclear cells (PBMCs) in a subset of Tampa COVID-19 survivors (n = 18) and an autopsied COVID lung tissue database was separately analyzed for biomarker gene expression.

**Results:**

Plasma biomarkers, including eNAMPT were significantly elevated in COVID-19 subjects compared to healthy controls (p < 0.0001), with IL-1RA, IL-6 and eNAMPT exhibiting the strongest diagnostic performance. Biomarker levels (all except IL-8, MIF) were higher in COVID-19 ARDS cases vs COVID-19 pneumonia (n = 154, AUC 0.71–0.79, p < 0.05) and were significantly elevated in COVID non-survivors (IL-6, IL-1RA, TNF-α, eNAMPT, ANG-2, PSGL-1) compared to survivors (p < 0.05). NAMPT gene expression was significantly upregulated in COVID-19 PBMCs compared with healthy controls, most evident within the CD14 + monocyte cluster. Relative to lung tissue from non-COVID-19 infected, NAMPT gene expression was increased in specific COVID-19 lung cell populations, including neutrophils, CD16 + monocytes, conventional dendritic cells (cDCs), fibroblasts, endothelium, and alveolar macrophages.

**Conclusion:**

Our study corroborated the utility of this biomarker panel for diagnostic and severity assessment in COVID-19- associated ARDS and as a strategy for subject stratification in therapeutic trials of eNAMPT-neutralizing therapies. We further identified NAMPT expression in immune effector cells that correlates with COVID-19 severity and lethality. Together, these findings suggest NAMPT plays a key role in sustaining inflammatory activation and impairing lung regeneration in fatal COVID-19.

## Introduction

The COVID-19 pandemic caused an unprecedented world-wide mortality largely driven by severe complications such as acute respiratory distress syndrome (ARDS) [1]. ARDS is a hyperinflammatory state marked by massive release of cytokines, pathogen–associated molecular pattern proteins (PAMPs), and damage-associated molecular pattern proteins (DAMPs) [2,3]. Despite advances in diagnostic and supportive care, there remains a significant unmet need for novel targeted pharmacotherapies and effective biomarkers of pathological severity and prognostic outcome.

Clinical studies have reported that elevated plasma and serum cytokine levels (IL-1β, IL-6, IL-8, TNF-α) in patients with severe COVID-19 have strong associations with mortality [4–6]. Similarly, in bronchoalveolar lavage fluid (BALF) elevated levels of the IL-1β antagonist (IL-1RA) were linked to the severity of COVID-19 pneumonia [7–9]. Our previous work in a limited cohort of COVID-19 positive subjects (n = 168) [3] additionally confirmed significant elevation of specific ARDS-associated plasma biomarkers (IL-6, IL-8, IL-1RA, ANG-2) potentially supporting their use in ARDS disease stratification.

In preclinical models of ARDS and ventilator induced lung injury (VILI) we have previously identified extracellular nicotinamide phosphoribosyl transferase (eNAMPT) as a damage-associated molecular pattern protein (DAMP) and the primary non-infectious ligand for Toll-Like Receptor 4 (TLR4) [10–12]. eNAMPT/TLR4 signaling induces NFκB transcription which amplifies innate immunity-driven systemic inflammation and multiorgan dysfunction [11–13]. Elevated eNAMPT has been documented across multiple critical diseases associated with ARDS including sepsis, trauma, pancreatitis, radiation pneumonitis and COVID-19 infection [14–18]. Studies have implicated linkage of plasma eNAMPT levels to ARDS mortality [13,17]. Preclinical mouse, rat and porcine ARDS/VILI studies demonstrated that an eNAMPT-neutralizing monoclonal antibody (mAb) significantly reduces acute lung injury as well as NFκB activation in lung tissues and blood [12,18–21]. ALT-100 mAb, a humanized monoclonal antibody designed to neutralize eNAMPT, has recently demonstrated excellent safety profiles. Succeeding in both a Phase 1a safety study involving healthy human volunteers and in a Phase 2a clinical trial in patients with ARDS (NCT05938036-PUERTA) [20] with interim analysis indicating clinical efficacy [22].

This set of plasma biomarker maps onto three converging arms of the fibrotic cascade relevant to COVID-19-associated ARDS: (1) cytokine-driven fibroblast/myofibroblast activation and matrix synthesis (IL-1β, IL-6, TNF-α, counterbalanced by IL-1RA), (2) DAMP-driven epithelial/endothelial-to-mesenchymal transition via TLR4/NF-κB and MAPK signaling (eNAMPT, MIF), and (3) endothelial/vascular destabilization and leukocyte-mediated tissue injury that sustains the remodeling (ANG-2, PSGL-1), with IL-8 more specifically reflecting acute neutrophilic injury.

The present study, we expand our prior findings by analyzing a comprehensive panel of inflammation and injury-related biomarkers (IL-6, IL-1β, IL-8, IL-1RA, TNF-α, eNAMPT, MIF ANG-2, PSGL-1) in a larger cohort of 277 hospitalized COVID-19 patients with the intent to potentially discriminate COVID-19 infection and predict pneumonia progression to ARDS and mortality. We further investigated the cellular sources and transcriptional profiles of biomarker genes using single-cell RNA sequencing (scRNA-seq) analysis of two independent datasets; peripheral blood mononuclear cells (PBMCs) from COVID-19 survivors and lung tissues from COVID-19 non-survivors. This integrative approach combining plasma protein levels and cellular gene expression, provides insight into eNAMPT’s involvement in innate immunity signaling underlying COVID-19 severity and demonstrates its potential utility in therapeutic trials stratification.

## Materials and methods

### Clinical cohorts

#### Ethics approval and consent to participate.

The clinical research studies for subjects consenting to participate were approved in each center by their respective Institutional Review Boards in compliance with the Declaration of Helsinki. Written informed consent was obtained from all participants, or their legally authorized surrogate for research purposes. IRB approvals included the University of Arizona’s Biorepository (IRB No.1712117680), the University of Miami (IRB No. 20150612) and the University of South Florida (IRB no. Pro00032158). This study utilized data and specimens with no identifiable information and met the criteria for nonhuman research authorized under University of Florida protocol # NH00049911. Data and samples were initially accessed for assay research purposes on 11/12/2024.

A total of 277 COVID-19 positive subjects were included in this study comprising cohorts from the University of Arizona (n = 145), the University of Miami (n = 61) and the University of South Florida (USF)/Tampa General Hospital (n = 71). Specimens were collected within two days (±2) from hospital admission. Inclusion criteria required a confirmed COVID-19 diagnosis by PCR or antigen test and the availability of hospitalization status information. ARDS was defined according to the Berlin criteria [23].

#### Plasma inflammatory biomarker quantitation.

A total of 491 plasma samples from healthy controls (n = 214) and COVID-19 positive patients (n = 277) were assayed for quantitation of diverse biomarker panel of inflammatory cytokines (IL-1β, IL-6, IL-8, IL-1RA, TNF-α), DAMPs (eNAMPT, MIF) and vascular proteins (ANG-2, PSGL-1). Plasma biomarker levels were quantified using a custom, multiplexed MSD (Meso Scale Discovery, MSD™) electrochemiluminescent detection platform as we previously reported [18,24].

#### Single Cell RNA sequencing (scRNA-seq).

We analyzed a previously published scRNA-seq dataset [25] from cryopreserved PBMCs deposited in GEO (GSE264196) comprising 18 samples that included healthy controls (n = 4), acute COVID-19 (n = 3), post–COVID-19 interstitial lung disease (post-COVID-19 ILD; n = 5), and idiopathic pulmonary fibrosis (IPF; n = 6). The primary analysis compared healthy controls with patients with acute COVID-19 to assess acute COVID-19-associated changes in circulating NAMPT expression. Post-COVID-19 ILD and IPF were considered clinically and biologically distinct chronic fibrotic disease states and were therefore included only in descriptive analyses across all four groups. We further utilized sequencing methods which we have described in previous studies [25]. Briefly, scRNA-seq data were processed in R using Seurat v5, normalized with SCTransform, and subjected to principal component analyses followed by UMAP dimensionality reduction. Transcriptionally distinct immune cell populations were identified using Louvain clustering and canonical marker-based annotation. To avoid pseudoreplication and account for unequal cell recovery, raw RNA counts were aggregated by donor and annotated cell type, normalized for library size, and compared between healthy controls and patients with acute COVID-19 using donors as biological replicates. Marker expression was visualized using DotPlots, FeaturePlots, and violin plots generated with Seurat, SeuratExtend, and ggplot2. Our biomarker panel was then interrogated in an independent publicly available dataset from The Integrated Human Lung Cell Atlas (HLCA) v1.0 generated from single nucleus RNA transcriptomics from COVID-19-infected lungs (n = 16) and control lungs (n = 7) [26] GEO accession number GSE162911. Data was analyzed using the interactive visualization tools from Single Cell Portal data [27].

#### Statistical analysis.

GraphPad Prism v. 10.4.2 (GraphPad Software, San Diego, CA, USA), was utilized to compute the statistical analyses conducted from both cohorts. Nonparametric tests were performed independently for both Discovery and Validation cohorts as they were found to not be normally distributed based on the D’Agostino & Pearson, Anderson-Darling, Shapiro-Wilk, and Kolmogorov-Smirnov normality tests. Receiver Operating Characteristic (ROC) tests and area under the curve (AUC) were performed to calculate discriminatory accuracy, sensitivity and specificity of the biomarker panel for diagnosis and disease severity progression Spearman correlations and linear regression were conducted for permutations of clinical parameters. Additional statistical analysis was performed in STATA 18.5 (StataCorp College Station, TX, USA), and R (R Core Team, 2020). Comparisons were performed across disease groups within clusters or cell types, non-parametric tests (Wilcoxon rank-sum for two groups; Kruskal–Wallis for multiple groups) were used with significance was displayed as star annotations.

## Results

### Demographics and phenotypes of COVID study cohorts

The demographic and clinical characteristics of healthy controls and the COVID-19 positive cohorts are summarized in Table 1. A total of 277 hospitalized COVID-19 patients were included in the study. Age differed significantly across cohorts, with the Miami cohort being the oldest (67.1 + 13.7 years), whereas the healthy controls were substantially younger than to all COVID-19 groups (42.8 + 16.9). To evaluate the relationship between age and circulating levels of the inflammatory and vascular biomarkers in COVID-19 subjects, we analyzed a subset of samples for eNAMPT and age bivariate correlation rho = 0.15, n = 163, p = 0.051, revealing a non-meaningful correlation (S1 Fig). Then a multiple variable regression, showed that the model overall was not statistically significant (F6,24) = 1.13, p = 0.377) and none of the biomarkers were independently associated with age, including eNAMPT (β = 0.157, p = 0.542), IL-1β (β = 2.352, p = 0.562), IL-1RA (β = 0.00046, p = 0.724), IL-6 (β = −0.061, p = 0.298), ANG2 (β = 0.00022, p = 0.553), and TNFα (β = 2.759, p = 0.406). These results indicate that the inflammatory and vascular biomarkers evaluated were not significant predictors of age in COVID-19. Thus, the observed elevations in inflammatory and vascular biomarkers are not explained by age differences within this cohort. The ratio of female to male was similar in all three cohorts, with no significant differences in the biomarkers levels (S2 Fig). All three COVID-19 cohorts were predominantly white. Assessment of the clinical variables associated with COVID-19 severity, indicated notable differences between sites, the Arizona cohort had the highest proportion of patients with pneumonia but not ARDS (53.8%), whereas the Miami cohort exhibited a greater number of patients progressing to ARDS (57.4%). Intubation was reported in 135 patients, 49%, with the Arizona cohort showing the highest intubation rate (66.2%). Mortality rates varied significantly among cohorts, at 27.6%, 39.3% and 18.3%, respectively, with the Miami cohort exhibiting the highest proportion and the Tampa cohort the lowest. Length of hospitalization also differed, with the Arizona cohort 1 reporting the longest average hospital stay (21.8 days), compared to 13.6 days in the Tampa cohort. ICU length of stay averaged 14 days in the Miami cohort 2 and 12.9 days in the Tampa cohort, further reflecting differences in disease severity across study sites.

**Table 1:** Demographic and Clinical Characteristics of the COVID-19 Cohorts.

	Healthy Controls	U. Arizona Cohort 1	U. Miami Cohort 2	U. South Florida Cohort 3	P value
**Subjects** (n)	214	145	61	71	
**Age** (Mean, SD)	42.8, 16.9	53.8, 14.4	67.1, 13.7	54.1, 15.8	<0.0001
**Sex** (Female %)	59.3	44.1	41	46.5	
**Race** (n, %)					
White	92 (43)	107 (73.8)	50 (82.0)	31(43.66)	
Black/African American	23 (10.7)	3 (2.1)	7 (11.5)	22(30.98)	
American Indian/ Alaska Native	1 (0.5)	19 (13.1)	0	1(1.40)	
Asian	5 (2.3)	4 (2.8)	0	15(21.12)	
N/A	93 (43.5)	12 (8.3)	4 (6.6)	2(2.81)	
**Ethnicity** (n, %)					
Hispanic or Latino	23 (10.7)	71 (49.0)	44 (72.1)	13(18.30)	
Non-Hispanic	49 (22.9)	62 (42.8)	15 (24.6)	57(80.28)	
**Lung Complication** (n, %)					
Pneumonia	—	78 (53.8)	16 (26.2)	60(84.50)	
ARDS	—	38 (26.2)	35 (57.4)	33(46.47)	
Other	—	29 (20.0)	10 (16.4)	6 (8.5)	
**Length of Stay** (n, mean)					
Hospital days	—	131 (21.8)	NA	69 (13.6)	<0.0001
ICU days	—	—	33 (14)	46(12.9)	0.4261
**Intubation** (n, %)	—	96 (66.2)	26 (42.6)	13 (18.3)	
**Mortality** (n, %)	—	40 (27.6)	24 (39.3)	13 (18.3)	
**Coagulation test** (n, mean + SD)					
PT	—	NA	48 (17.78, 17.83)	26 (18.16, 18.2)	0.4
INR	—	NA	48 (1.450, 1.614)	26(1.5, 1.5.48)	0.97
PTT	—	NA	48 (46.58, 39.94)	18(37.7, 25.6)	0.23

N/A non-available; Intensive care unit (ICU); Prothrombin time (PT); International Normalized Ratio (INR); Partial Thromboplastin Time (PTT). Kruskal-Wallis test.

### Association of eNAMPT- ANG-2 and Endothelial/ Tie-axis injury

ANG-2, is a marker of endothelial-axis injury that disrupts the Tie2 signaling, promoting vascular permeability, driving EC dysfunction, inflammation, and lung injury [28,29]. Using a subset of samples with paired measurements we analyzed the eNAMPT-ANG-2 association. Spearman correlation using the five markers (eNAMPT, ANG-2, IL-6, IL1RA, IL-1β), commonly measured across most of our samples demonstrated that eNAMPT is significantly correlated with all four markers, with IL-1RA is correlation being the strongest (rho = 0.53, n = 165, p < 0.0001), followed by IL-6 (rho = 0.45, n = 160, p < 0.0001) and IL-1β (rho = 0.41, n = 33, p = 0.02). The correlation between eNAMPT and ANG-2 although the weakest is still statistically significant (rho = 0.24, n = 164, p = 0.0021) (S3 Fig) reflecting a shared covariation within the broader inflammatory response. When we additionally modeled eNAMPT in a multivariable regression including IL-6, IL-1β, and IL-1RA, ANG-2’s association was no longer significant (p = 0.88) indicating that ANG-2’s effect is lost once IL-6 is accounted for. These results suggest that eNAMPT and ANG-2 elevation largely co-occur as part of a shared, IL-6-driven systemic inflammatory response rather than reflecting a direct, independent biological interaction between eNAMPT and endothelial/vascular, supporting their combined inclusion in our biomarker panel.

#### Biomarkers associated with COVID-19 disease severity and mortality.

Biomarker comparisons across the three cohorts showed significantly higher levels of ANG-2 and IL-6 in Miami cohort 2, and significantly higher levels of IL1RA and eNAMPT in Arizona cohort 1. Different biomarkers were available for different cohorts, i.e., IL-1β, PSGL-1 and TNFα were only measured in the Miami cohort 2 and Tampa cohorts 3 and were significantly higher in the Miami cohort 2 (S1 Table). To correct for these significant batch effects, the remaining analysis was performed in a COVID-19 merged set (Table 2). Plasma concentrations of each biomarker were significantly elevated in the COVID-19 group (Fig 1A), **except for PSGL-1,** with the strongest discriminatory performance based on AUCs exhibited by three biomarkers:; IL-1RA (AUC 0.9, p=<0.01, CI: 0.93 to 0.97), IL-6 (AUC 0.94, p= < 0.01, CI: 0.92 to 0.96), NAMPT (AUC 0.85 p=<0.0 CI:0.82 to 0.888). Moderate discriminatory capacity (AUC > 0.70, p < 0.001) was observed for ANG-2 (CI 0.714 to 0.978):, TNF-α (CI 0.73 to 0.85), IL-1β (CI: 0.63 to 0.77), IL-8 (CI 0.76 to 0.87), and MIF (CI: 0.74 to 0.87). Finally, PSGL-1 (CI: 0.56 to 0.72) was the marker with the lowest AUC (0.64, p = 0.03) (Fig 1B).

**Table 2:** Healthy Controls and COVID-19 Biomarkers Levels.

	Healthy Controls	COVID-19	P-value
**eNAMPT**	209, 2.2 (3.8-1.2)	277, 8.1 (16.2–3.8)	<0.0002
**IL-1RA**	201, 105.3 (183.1-66.5	277, 758 (2234.2-375.5)	<0.0001
**IL-1β**	111, 0.1 (0.2-0.1)	82, 0.3 (0.5 - 0.1)	<0.0001
**IL-6**	199, 0.7(1.2–0.4)	272, 6.4 (17.8 - 2.9)	<0.0191
**IL-8**	65, 4.2 (5.8–2.9)	145, 11.5 (29.5 - 5.3)	<0.0001
**ANG-2**	213, 2079 (3244.3-951.2)	275, 4484.4(8075.0-2327.3)	<0.0001
**MIF**	45,16306.3(23165.3-11048.1)	145, 44311.1(61475.3-21435.4)	<0.0001
**PSGL-1**	53, 5000.6(6176.7- 4082.4)	132, 3954.5 (5770.4-2761.5)	<0.0001
**TNF-α**	77, 0.9 (1.2- 0.6)	124, 1.6 (2.9 −1.1)	<0.0001

Statistics of biomarkers assayed (number of samples, median, upper limit, lower limit quartile). Extracellular NAMPT (eNAMPT), Interleukin 1 receptor antagonist (IL-1RA), Interleukin 1 beta (IL-1β), Interleukin 6 (IL-6), Interleukin 8 (IL-8), Angiopoietin 2 (ANG-2). Mann-Whitney tests were performed to assess significance between healthy controls vs COVID-19 positive subjects.

**Fig 1:**
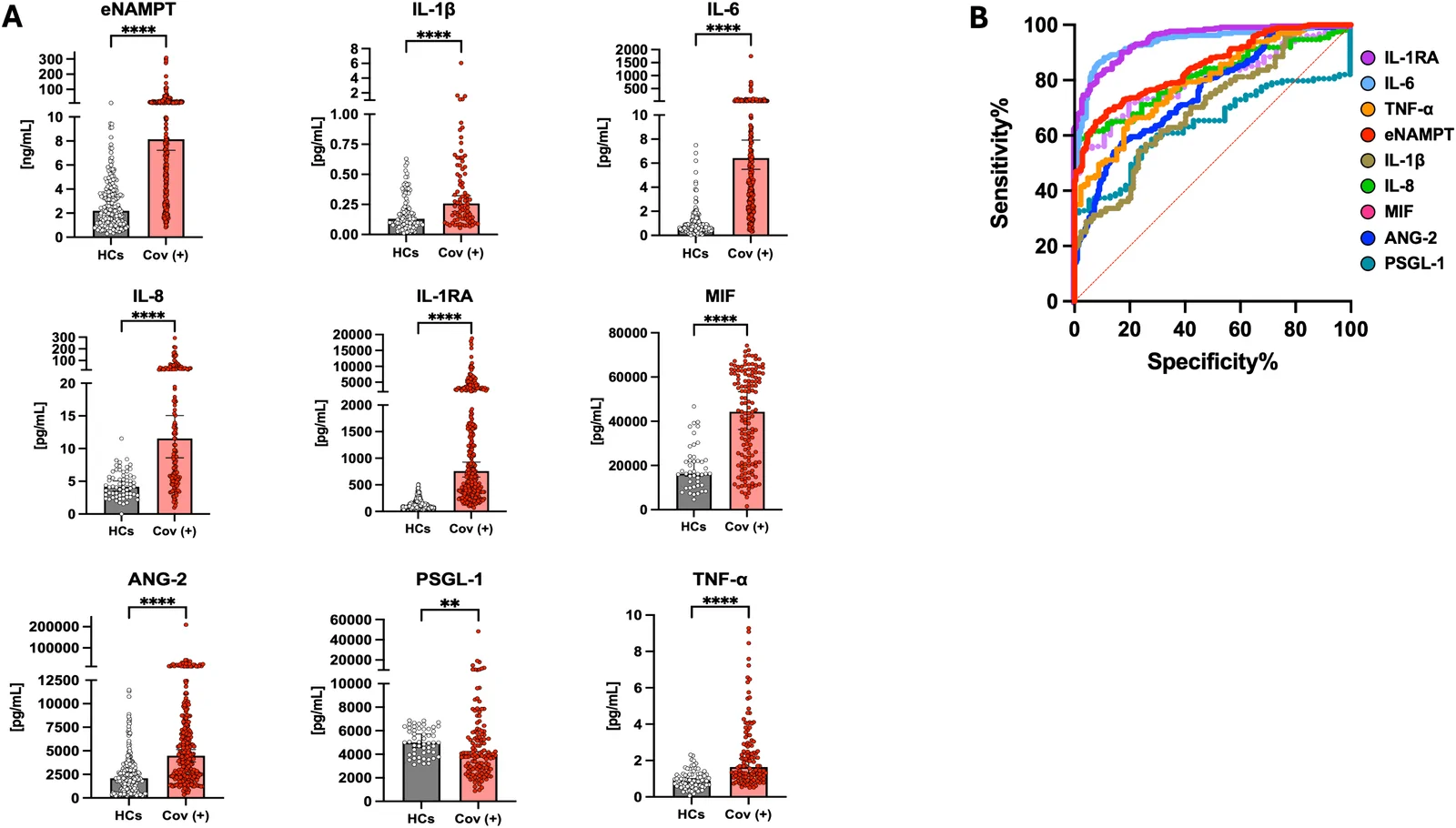
Comparison of plasma biomarker levels in healthy controls (n = 209) and COVID-19. **A.** healthy controls (n = 209) and COVID-19 positive patients (n = 277). Due to technical differences in the biomarker panel over time, MIF and IL-8 were assessed exclusively in Arizona (cohort 1) while TNF-α, IL-1β and PSGL-1 were assessed only in Miami (cohort 2) and the Tampa (cohort 3). Total readings analyzed per biomarker for the healthy control cohort and all COVID-19 cohorts respectively were: eNAMPT (n = 209 vs. 277), IL-1β (n = 111 vs. 82), IL-6 (n = 199 vs. 272), IL-8 (n = 65 vs. 145), IL-1RA (n = 201 vs. 277), MIF (n = 45 vs. 145), ANG-2 (n = 213 vs. 275), PSGL-1 (n = 53 vs. 132), and TNF-α (n = 77 vs. 124). Results from all-merged COVID-19 analysis showed all biomarkers, except PSGL-1 were significantly elevated compared to healthy controls (p < 0.0001 for each). Although PSGL-1 levels were not significantly elevated in COVID-19, compared to controls, it showed a subsequent increase in ARDS **B.** Area Under the Curve of Receiver Operating Curve (AUC-ROC) plots of healthy controls vs COVID-19 separating the true positive rate presented as Sensitivity % (Y axis) vs the false positive rate, showed as Specificity % (X-axis). Indicated a strong positive predictive value with AUC: 0.76–0.97 range among all 8 significant analytes. Strongest discriminatory power based on AUCs were observed for three markers: IL-1RA AUC 0.9 (p=<0.01, CI: 0.93 to 0.97), IL-6 AUC (p= < 0.01, CI: 0.92 to 0.96), NAMPT AUC 0.85 p=<0.0 CI:0.82 to 0.888). Moderate discriminatory capacity for ANG-2 (AUC 0.75, p value<0.00, CI: 0.71 to 0.98), TNF-α (AUC 0.79, IL-1β, Il-8, and MIF. PSGL-1 was the marker with the lowest AUC 0.64 (p= < 0.03, CI).

Analysis for progression of COVID-19 severity from pneumonia to ARDS showed significant elevation in eNAMPT, IL-6, IL-8, ANG-2, PSGL-1, and TNF-α (>1.6-fold change) in ARDS cases compared to pneumonia subjects. Plasma levels of MIF, IL-1 β and IL-8 trended higher in ARDS but were not significant (Fig 2A). TNF-α, PSGL-1 and IL-6 showed the highest diagnostic accuracy, TNF-α, (AUC 0.79, p = 0.0009, CI: 0.67 to 0.91), PSGL-1 (AUC 0.75, p = 0.0045, CI: 0.662 to 0.88) and IL-6 (AUCs 0.71, p value<0.0001, CI: 0.63 to 0.79) (Fig 2B). Analysis of the requirement for intubation and ventilatory support as another index of disease severity confirmed significant elevations in eNAMPT, PSGL, IL-6, IL-1RA, TNF-α and IL-8 (p < 0.05) compared to those that did not required intubation for ventilatory support (Fig 2C). Last the eNAMPT odds ratios in ARDS vs pneumonia and survivors vs non-survivors were calculated using two different models, one unadjusted and another one adjusted for all biomarkers, age and gender. Higher eNAMPT was associated with significantly higher odds of having ARDS rather than pneumonia alone. A one-log-unit increase in eNAMPT was associated with approximately 44–50% increase in the odds of ARDS in adjusted (p = 0.013, CI 1.08–1.95) and unadjusted (p = 0.0039, CI 1.15–2.0) models (Fig 3D).

**Fig 2:**
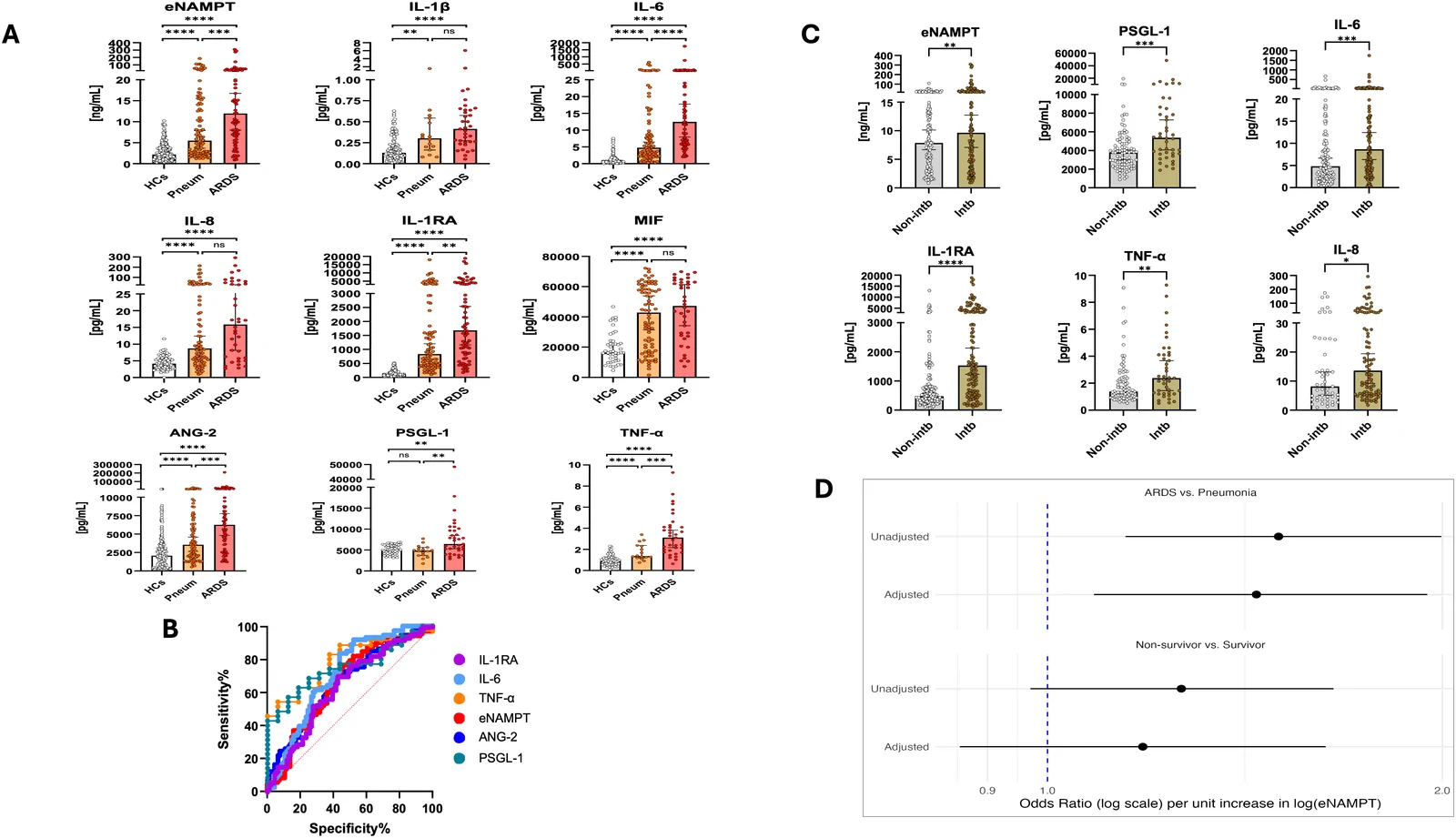
COVID-19 biomarker severity assessment, differences between pneumonia and ARDS. **A.** COVID-19 infection severity was evaluated by comparing the biomarker circulating levels in pneumonia (Pneum) (n ≈ 94) vs those that developed ARDS (n ≈ 73). Significantly higher levels in eNAMPT, IL-6, IL-8, IL-1RA, ANG-2, PSGL-1, and TNF-α were present in ARDS compared to subjects with limited pneumonia (p < 0.05). MIF, IL-1 β and IL-8 showed no significant difference. **B.** Evaluation of the diagnostic accuracy of the biomarker levels between pneumonia and ARDS was performed calculating the AUC of ROCs. TNF-α, PSGL-1 and IL-6 exhibited the highest discriminatory power. TNF-α, (AUC 0.79, p = 0.0009, CI: 0.67 to 0.91), PSGL-1 (AUC 0.75, p = 0.0045, CI: 0.662 to 0.88) and IL-6 (AUCs 0.71, p value<0.0001, CI: 0.63 to 0.79). **C.** Reported intubation status was also assessed for an approximate total of 139 intubated (int) and 138 non-intubated (Non-intb). Subjects intubated and receiving mechanical ventilation exhibited significantly (p < 0.05) elevated biomarker levels (eNAMPT, IL-1RA, PSGL, IL-6, TNF-α and IL-8) compared to non-intubated subjects. **D.** eNAMPT odds ratios in ARDS vs pneumonia and survivors vs non-survivors. Comparison between unadjusted and adjusting for age, sex and all biomarkers. Higher eNAMPT is associated with significantly higher odds of having ARDS rather than pneumonia alone. A one-log-unit increase in eNAMPT is associated with about a 44–50% increase in the odds of ARDS in adjusted (p = 0.013, CI: 1.08-1.95) and in unadjusted (p = 0.0039, CI: 1.15–2.0) models. In non-survivors there was a trend toward higher eNAMPT association with mortality but did not achieve statistically significant in either model (p > 0.3).

**Fig 3:**
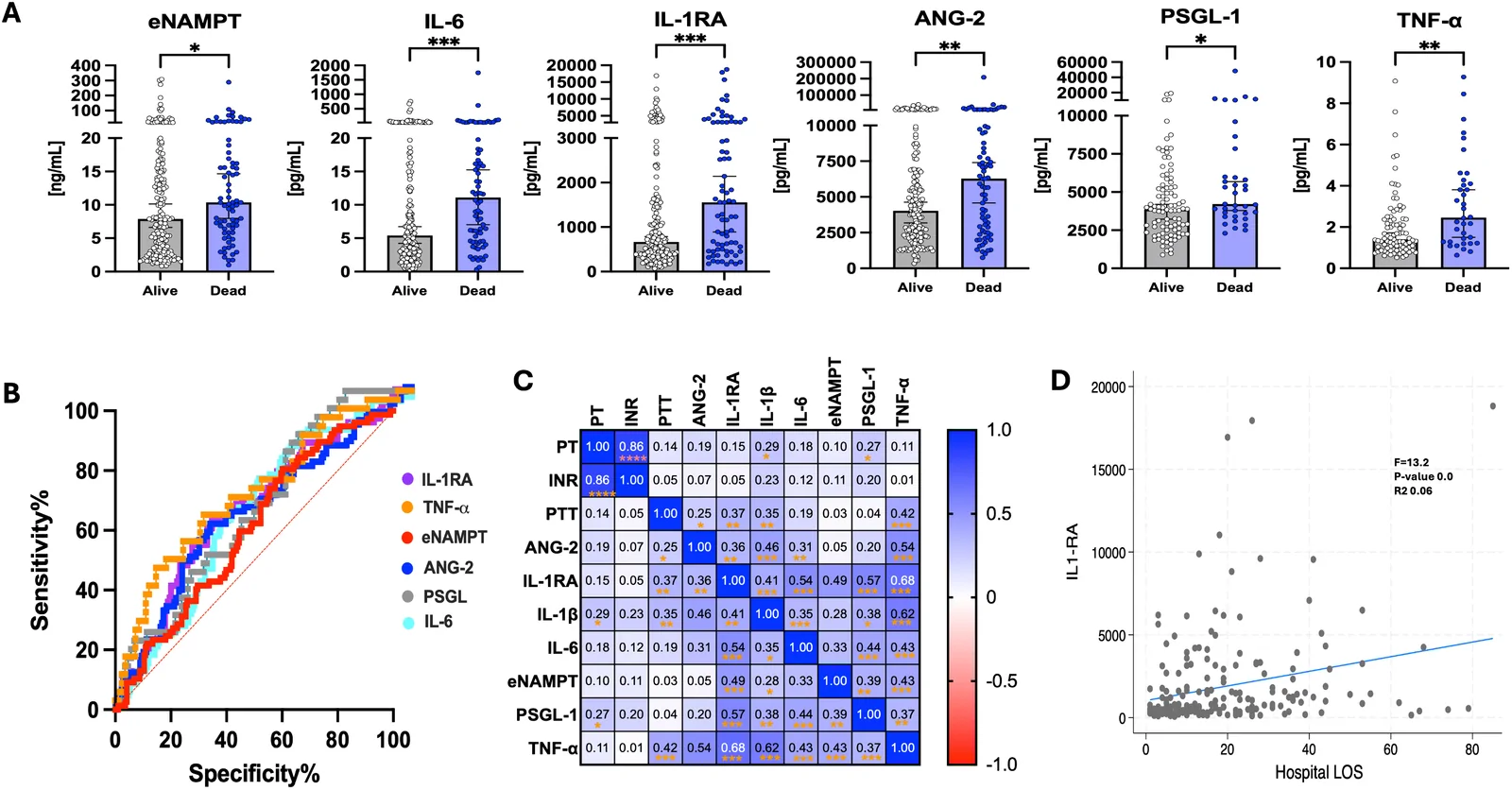
Association of biomarker levels with COVID mortality. **A.** The levels of eNAMPT (p < 0.04) IL-6 (p < 0.04), IL-1RA (p < 0.04), ANG-2 (p < 0.04), PSGL-1 (p < 0.05) and TNF-α (p < 0.05) were significantly elevated in non-survivors (medians with 95% CI). **B.** AUCs show a moderate capability of predicting mortality of 0.66–0.70 among the significantly elevated analytes. **C.** Coagulation parameters, INR, prothrombin time (PT) and activated partial thromboplastin time (PTT), were assessed by Spearman correlation. PT was significantly positive correlated with IL-1β (rho 0.3 CI: 0.3-0.5) and PSGL-1 (rho 0.27, CI: 0.3-0.4). PTT showed a positive correlation with ANG-2 (rho 0.25, CI:0.03-0.5), IL-1RA (rho 0.37, CI:0.13 - 0.56), IL-1β (rho 0.35, CI: 0.09 - 0.06) and TNF-α (rho 0.4, CI: 0.19-0.6). INR was not correlated with any of the biomarkers in this panel. These results confirm the association of the COVID-19-associated coagulopathy with inflammatory biomarkers. **D.** Correlation of the plasma biomarkers with hospitalization length of stay (LOS) found only IL-1RA with a weak but significant positive linear correlation (p < 0.01; r^2^ = 0.06; regression coefficient = 0.0014) as the variation remains unexplained by this single predictor (standard error of the estimate: Sy.x = 15.6).

Non survivors show levels of eNAMPT (p < 0.04) IL-6 (p < 0.04), IL-1RA (p < 0.04), ANG-2 (p < 0.04), PSGL-1 (p < 0.05) and TNF-α (p < 0.05) significantly elevated compared to survivors (Fig 3A). Analyses of the discriminatory accuracy of plasma biomarkers levels for survival revealed only a modest to prediction of mortality in six significantly elevated biomarkers: TNF-α (p < 0.01; AUC = 0.70), PSGL-1 (p < 0.02; AUC = 0.64), eNAMPT (p < 0.02; AUC = 0.61), IL-6 (p < 0.01; AUC = 0.63), IL-1RA (p < 0.05; AUC = 0.64), and ANG-2 (p < 0.01; AUC = 0.61) (Fig 3B). In non-survivors we observed a trend toward higher eNAMPT being associated with higher odds of death, however, it was not statistically significant (adjusted model: OR 1.18 p > 0.3, unadjusted: 1.3 p = 0.08) (Fig 3D).

As a final index of severity, the correlation of plasma biomarkers with hospitalization length of stay (LOS) was assessed, demonstrating a significant association with IL-1RA, a positive though weak, linear correlation (p < 0.01; r [2] = 0.06; regression coefficient = .0014) indicating most of the variation is not explained by this single predictor (Standard Error of the Estimate: Sy.x = 15.6). Since coagulation abnormalities are a significant contributor to morbidity and mortality in COVID-19 [25], we additionally analyzed the linkage of biomarker levels to prothrombin time (PT), international normalized ratio (INR), and activated partial thromboplastin time (PTT). There were no significant differences in PT, INR, or activated PTT between the three cohorts (Table 1), however, an association identified between PT and IL-1β (rho 0.3 CI: 0.3–0.5) and PSGL-1 (rho 0.27, CI: 0.3–0.4) and between PTT and ANG-2 (rho 0.25, CI:0.03–0.5), IL-1RA (rho 0.37, CI:0.13–0.56), IL-1β (rho 0.35, CI: 0.09–0.06) and TNF-α (rho 0.4, CI 0.19–0.6) (Fig 3C).

#### Increased NAMPT expression in COVID-19 CD14 monocytes.

To identify potential cellular sources of circulating *NAMPT*, we analyzed previously published PBMC scRNA-seq data from healthy controls, patients with acute COVID-19, individuals with post-COVID-19 ILD, and patients with IPF (GSE264196; S2 Table). Following preprocessing, dimensionality reduction, clustering, and canonical marker-based annotation, 22 transcriptionally distinct clusters representing 13 major immune populations were identified, including age-associated B cells, effector and early-effector CD8 T cells, naïve CD4 and CD8 T cells, CD14⁺ and CD16 ⁺ monocytes, naïve B cells, platelets, plasmacytoid dendritic cells, cDC2 cells, proliferating cells, and plasmablasts (Fig 4A). Cell-composition analysis demonstrated marked donor-to-donor heterogeneity, particularly within the acute COVID-19 group, which also contained fewer recovered cells than the healthy-control group (Fig 4B).

**Fig 4:**
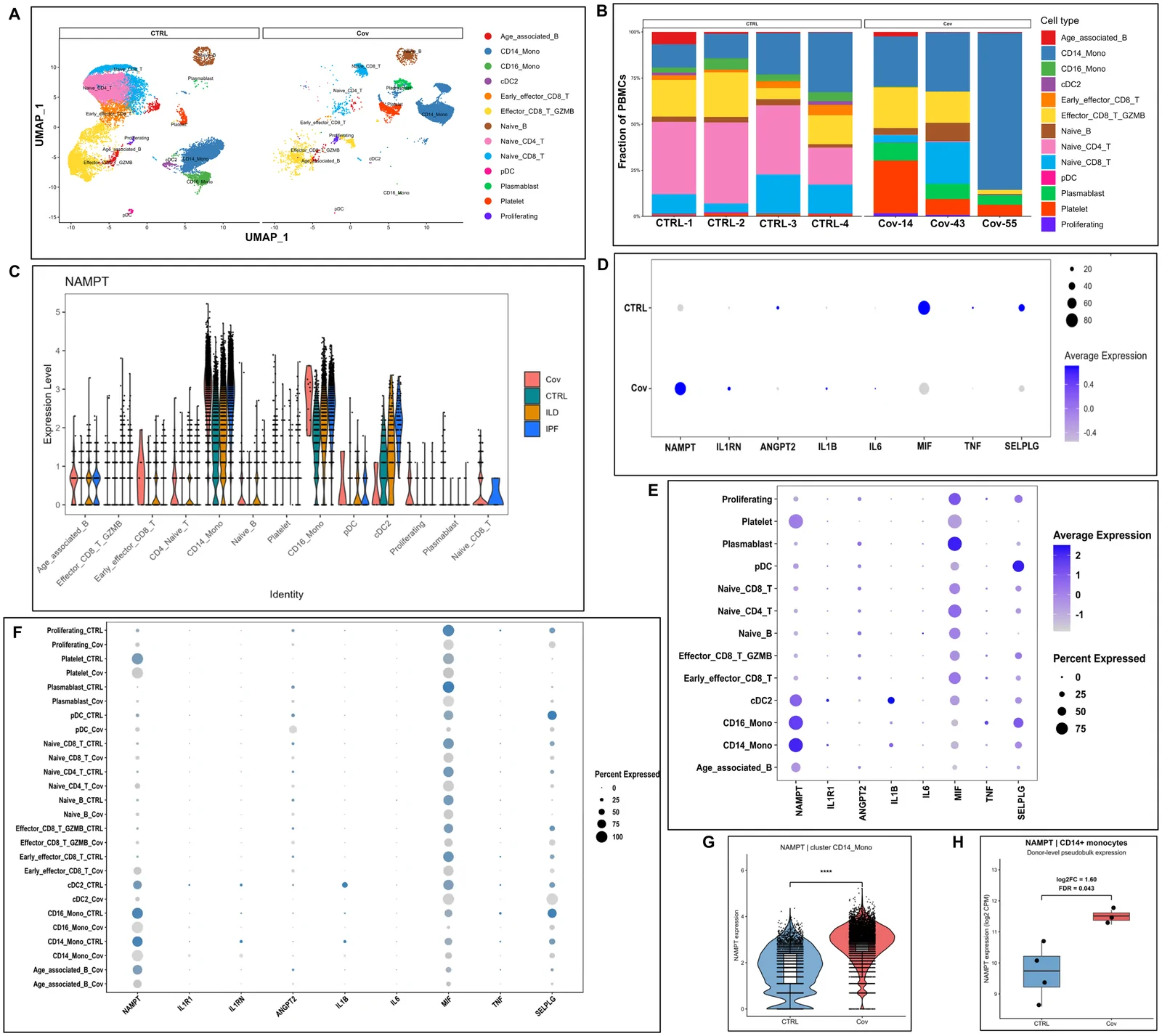
PBMC single-cell transcriptomic analysis in healthy controls and patients with acute COVID-19. **A.** UMAP visualization of annotated peripheral blood mononuclear cell populations from healthy controls (CTRL) and patients with acute COVID-19 (Cov), displayed separately by clinical group**. B.** Relative abundance of annotated PBMC populations in each donor, with healthy controls presented first. **C.** Violin plots showing NAMPT expression across immune-cell populations in healthy controls, acute COVID-19, post-COVID-19 interstitial lung disease (ILD), and idiopathic pulmonary fibrosis (IPF); the ILD and IPF groups were included for descriptive comparison only. **D.** DotPlot showing NAMPT, IL1R1, IL1RN, ANGPT2, IL1B, IL6, MIF, TNF, and SELPLG expression across the major PBMC populations, with dot size representing the percentage of biomakers-expressing cells and color indicating average normalized expression. **E**. DotPlot showing the expression of NAMPT and selected inflammatory biomarker genes, including IL1R1, IL1RN, ANGPT2, IL1B, IL6, MIF, TNF, and SELPLG, across annotated PBMC populations. Dot size represents the percentage of expressing cells, and color indicates scaled average expression. **F**. Direct comparison of biomarker-gene expression between healthy controls and patients with acute COVID-19 across corresponding cell populations. **G**. Cell-level violin and boxplot showing increased NAMPT expression in CD14 ⁺ monocytes from patients with acute COVID-19 relative to healthy controls. **H**. Donor-level pseudobulk analysis of NAMPT expression in CD14 ⁺ monocytes. Raw RNA counts were aggregated by donor and cell type, normalized for library size, and analyzed using donors as independent biological replicates. NAMPT expression was significantly increased in acute COVID-19 relative to healthy **controls (log_2_ fold change = 1.60; FDR = 0.043).**

Across all four clinical groups, *NAMPT* expression was concentrated predominantly within the myeloid compartment, with the highest expression observed in CD14⁺ and CD16 ⁺ monocytes relative to lymphoid populations (Fig 4C–4E). Because post-COVID-19 ILD and IPF represent clinically and biologically distinct chronic fibrotic states, the principal differential comparison was restricted to healthy controls and patients with acute COVID-19. Direct cluster-level DotPlots showed the distribution of *NAMPT* and related inflammatory genes across corresponding immune populations in the two groups, with the most prominent COVID-19-associated *NAMPT* signal occurring in the monocyte compartment (Fig 4F).

Cell-level boxplot analysis showed increased *NAMPT* expression within the CD14 ⁺ monocyte cluster (Fig 4G). Given the unequal cell recovery between groups, formal differential-expression testing was also performed using donor-level pseudobulk profiles. We observed significantly higher *NAMPT* expression in CD14 ⁺ monocytes from patients with acute COVID-19 relative to healthy controls, with a log2 fold change of 1.60 and an FDR of 0.043 (Fig 4H). Collectively, these findings indicate that acute COVID-19 is associated with a selective increase in *NAMPT* expression within circulating CD14 ⁺ monocytes rather than a uniform elevation across all PBMC lineages.

#### PBMC expression of biomarkers genes.

Within the monocyte compartment, higher *NAMPT* expression coincided with expression of biomarker genes (*IL1B, TNF, MIF, IL1RN/IL1RA, SELPLG)*. Among all biomarker genes, *NAMPT* expression was the most upregulated in CD14 + monocytes in COVID-19 relative to healthy controls. Consistent with the plasma biomarker findings, MIF and PSGL-1, encoded by SELPLG, were also increased at the transcript level, particularly in plasmacytoid dendritic cells. Together, these data identify circulating CD14 ⁺ monocytes as the potentially an important cellular source of increased NAMPT expression in acute COVID-19 and demonstrate coordinated upregulation of NAMPT-associated inflammatory markers across selected PBMC populations (Fig 4D–F).

#### Upregulated NAMPT expression in autopsied COVID lung tissues.

COVID-19 infected lung tissue primarily demonstrated a generalized increased expression of AT1, AT2, CD4 T cell, CD8 T cell and CD 16 monocyte among the top predicted cell types compared to control [30 30] (Fig 5A). Consistent with PBMC findings, *NAMPT* expression was highly upregulated in lung tissues from COVID-19 non-survivors (GSE#162911)^31^, relative to healthy lung tissue, with NAMPT expression increased in neutrophils, CD16 + T cells, monocytes, cDCs, and fibroblasts (Fig 5B). Gene expression of the circulating biomarkers measured in plasma was assessed *(NAMPT, ANG-2, IL1B, IL-6, MIF, TNF*- α, SELPLG and *IL-1RA/IL1RN) in* COVID-19 lung tissues, showing the cell-specific gene expression. Among all the biomarkers measured in this panel, *NAMPT* was the most upregulated of the biomarkers expressed in these COVID-19 non-survivor lung tissues (Fig 5C) and in multiple immune cell subphenotypes (neutrophils, CD16 monocytes, CdC, fibroblasts, globet cells and macrophages) but also when we contracted the expression across those virally infected tissues. ANG-2 was over expressed in lymphatic endothelial cells, while MIF was elevated in basal; and NKT cells. The remaining of the biomarkers did not showed significant differences.

**Fig 5:**
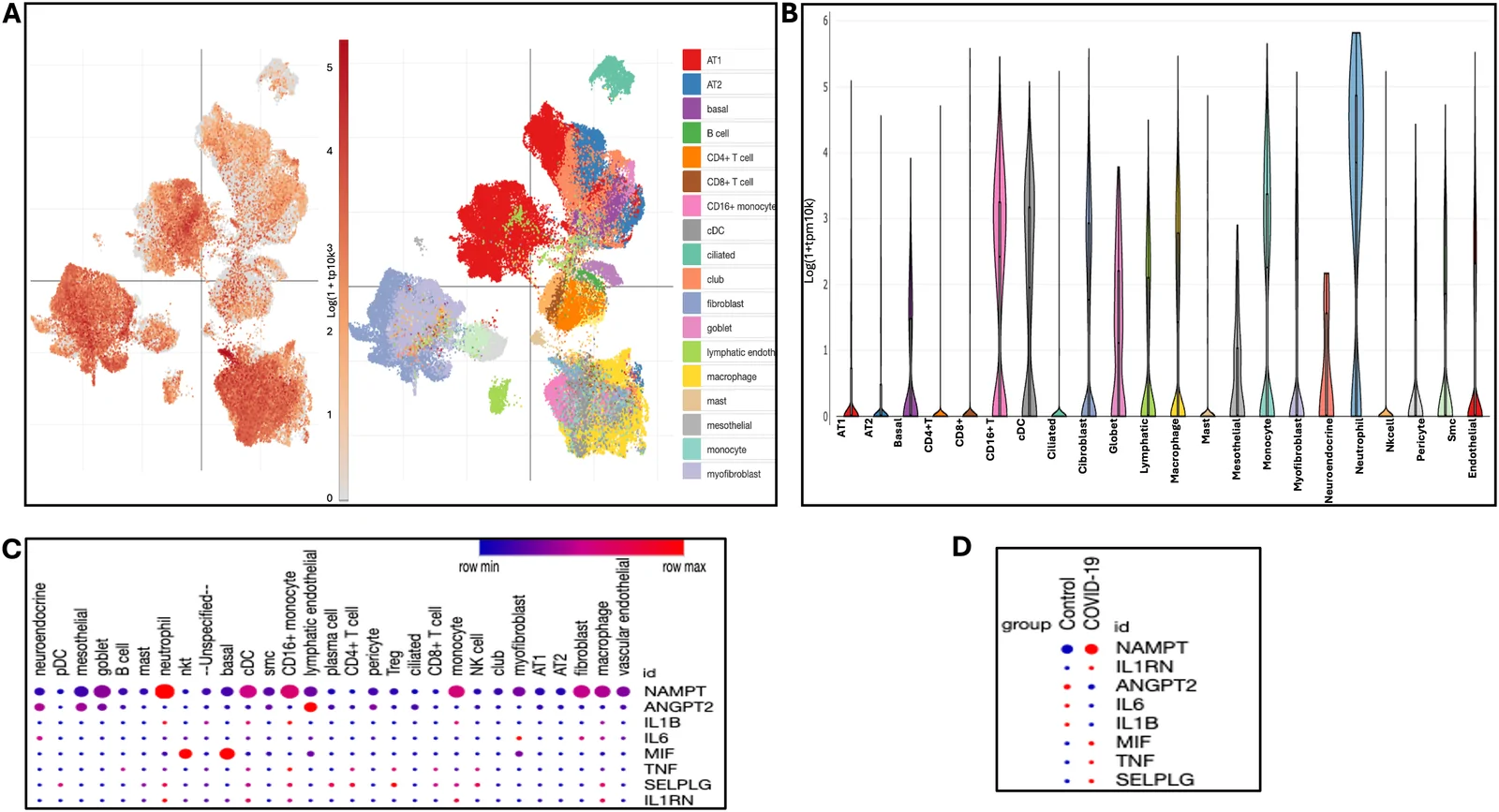
*NAMPT* gene expression in single cell RNA sequencing from lung tissue of COVID-19 positives and COVID-19 negatives. **A.** Matrix and UMAP (Uniform Manifold Approximation and Projection) with transcriptomes from lung autopsies COVID-19 (GEO #GSE171668) were obtained from the Single Cell portal [25] with the cell subtypes represented by the color code on the right, cells with the highest expression are plotted on top in descending order. AT1, AT2, CD4 T cell, CD8 T cell and CD 16 monocyte were among the top predicted cell types in COVID-19 lung tissue infected compared to control**. B** NAMPT expression distribution by cell type. The highest expression was observed in neutrophils, monocytes and CD16 monocytes. **C**. Dot plot of scaled, log-normalized expression of each of the circulating biomarkers *NAMPT, ANG-2, IL1B, IL-6, MIF, TNF*- α, SELPLG and *IL-1RA/IL1RN,* in COVID-19 lung tissues, showing the cell-specific gene expression, the dot size indicates the percentage of cells per cell subtype. Elevated NAMPT expression indicated in neutrophils followed by CD16, cCD fibroblasts and macrophages **D.** COVID-19 viral positive vs non-COVID-19 infected, dot plot of scaled, log-normalized expression, of the biomarker’s measures in plasma, plots are sorted by cells with the highest expression.

## Discussion

The biomarker panel in this study was subjectively selected to incorporate insights of biological processes that are relevant during lung inflammation and ARDS development. L-6, IL-1β, IL-1RA, eNAMPT, and ANG-2 are key mediators linking inflammation to fibrosis. IL-6 and IL-1β promote NF-κB activation, immune cell recruitment, and fibroblast activation, while eNAMPT amplifies inflammatory signaling as a damage-associated molecular pattern. Elevated IL-1RA reflects a compensatory response to ongoing inflammation, and ANG-2 contributes to endothelial dysfunction and vascular leak [29]. Together, these biomarkers quantify persistent inflammatory and vascular injury pathways that drive fibrotic remodeling observed in COVID-19 progression. This plasma biomarker panel demonstrated robust diagnostic accuracy in COVID-19 subjects with several biomarkers showing high discriminatory performance: IL-6, IL-1RA and eNAMPT (AUC > 0.85, p < 0.0001) thus reinforcing the utility of circulating inflammatory mediators as effective indicators of COVID-19 infection. Important specific elements of the panel also exhibited significant prognostic value in COVID-19 with elevated eNAMPT, IL-6, IL-1RA, ANG-2, and TNF- α levels linked to progression from limited pneumonia to ARDS, the need for mechanical ventilation, and with mortality. Hospital LOS data was reported in two cohorts with only IL-1RA having a significant positive correlation and significant linear correlation for hospitalization LOS. Nevertheless, the strong association of our biomarker panel with clinical outcomes underscores their potential as prognostic tools.

Our merged biomarker analyses reinforce the concept that hyperinflammatory, endothelial-injury endotype characterizes a subset of COVID-19 with high risk for progression to ARDS and death. We observed that the biomarker levels differ significantly across the three cohorts, with higher levels of ANG-2, IL-6, IL-1β, PSGL-1 and TNFα present in the Miami set; that was consistent with greater disease severity of this cohort (highest ARDS incidence, mortality and longer ICU length of stay). Our merged biomarker analyses reinforced that hyperinflammatory, endothelial-injury endotype characterizes a subset of COVID-19 with high risk for progression to ARDS and death reports on COVID-19 studies showing increased levels of IL-1β, IL-6, IL-8, and TNF-α in plasma and serum [4–6], and elevated IL-1RA in bronchoalveolar lavage fluid (BALF) of patients with COVID-19-induced pneumonia [7,9], in addition to association of IL-1β, IL-6, TNFα with worse clinical course and prognosis [31–33]. Although PSGL-1 levels were not significantly elevated in COVID-19, and showed an inversed trend, when compared to controls, PSGL-1 levels were significantly elevated in ARDS and showed a good discriminatory performance when differentiating ARDS from pneumonia, supporting published reports in COVID-19 subjects [34]. Our combined use of this biomarker panel has demonstrated to have a specific value on measuring disease progression. Since no single marker fully predicts severity, combining orthogonal signals (inflammatory, DAMP, vascular) recovered discriminatory power for severity-stratification stage.

In our study eNAMPT emerged as a biomarker with particularly strong relevance to COVID-19 severity and outcomes. Plasma eNAMPT levels demonstrated a high diagnostic accuracy achieving AUC over 0.85 with a strong significance distinguishing COVID-19 patients from healthy controls. Beyond diagnostic utility, eNAMPT remained remarkably elevated in patients who progressed to ARDS compared with those with limited pneumonia, and in those that required intubation for ventilatory support. Importantly, when mortality was assessed, eNAMPT levels were higher in non-survivors. indicator of evolving hyperinflammation and respiratory deterioration. These findings reinforce eNAMPT as a central component of the hyperinflammatory-endothelial injury phenotype observed in COVID-19 and highlight its utility as a biomarker for severity stratification and mortality risk assessment.

scRNA sequencing results on PBMCs from COVID19 survivors and in autopsied lung tissues from COVID19 non-survivors revealed potential cellular sources of circulating eNAMPT levels with *NAMPT* expression high in mononuclear cell population of CD14 + monocytes and in CD16 + monocytes. While biomarker gene expression across PBMC subsets was relatively stable, *NAMPT* expression was the biomarker gene with the most pronounced transcriptional upregulation in inflammatory cell population, a finding consistent with its role as a major TL-4-binging DAMP. These results also indicate the macrophage acquisition of transcriptional signatures reflecting profibrotic macrophages identified in idiopathic pulmonary fibrosis (IPF) [35]. These results are consistent with the observed macrophage and mesenchymal cell responses, and extensive fibrotic tissue remodeling, indicating an exacerbated fibroproliferative response in COVID-19-associated ARDS [36]. scRNA-seq analysis of autopsied COVID-19 lung tissue showed increased NAMPT expression specifically within the endothelial cell population, as well as neutrophils, CD16 + monocytes, cDCs, fibroblasts, and alveolar macrophages. Indicating that pulmonary endothelium is not only a target of eNAMPT-driven inflammatory injury but also a potential contributing source of circulating eNAMPT during severe COVID-19, a dual role consistent with the broader literature on endothelial cells as both effectors and amplifiers of DAMP-driven inflammatory injury and supported by our work utilizing the conditional endothelial cell eNAMPT KO mouse exposed to ARDS stimuli [20]

While our findings do not definitively identify all cellular sources of circulating eNAMPT, the combined analysis of systemic immune cells and pulmonary cell populations provides biologically plausible cellular candidates particularly circulating monocytes and specific pulmonary cell populations that include the endothelium. Finally, we acknowledge that circulating protein concentrations cannot be directly inferred from mRNA expression levels measured by scRNA-seq. Consistent with its role as a general DAMP/TLR4 ligand rather than a pathogen-specific marker, eNAMPT is proposed as a general marker of ARDS pathobiology with demonstrated utility in COVID-19-associated ARDS specifically, rather than as a COVID-19-specific biomarker

We acknowledge multiple limitations in our study that includes the modest sample size, heterogeneity among cohorts, incomplete harmonization of outcome reports (UCI vs hospitalization length of stay), and missing biomarker measurements across the cohorts (i.e., MIF, TNF- α, PSGL-1, IL-8). We further acknowledge the limitations associated with the lack of paired circulating biomarker and single cell transcriptomic data from the same patient cohort. Additionally, the small number of acute COVID-19 donors and the lower cell recovery relative to healthy controls. Next, although donor-level pseudobulk analysis minimizes pseudoreplication and is less sensitive to unequal cell numbers, the limited sample size reduces statistical power, particularly for rare PBMC populations. Furthermore, the findings were also not validated in an independent scRNA-seq cohort. Finally, we acknowledge that circulating protein concentrations cannot be directly inferred from mRNA expression levels measured by scRNA-seq.

## Conclusion

In summary, this study confirms a panel of biomarkers associated with diagnostic and severity in COVID-19. The combined analysis of systemic immune cells and pulmonary cell populations provides biologically meaningful evidence regarding candidate cellular contributors to the observed biomarker profile during COVID-19 infection. Our study identified eNAMPT as a biomarker of COVID-19 severity across three cohorts. The integration of independent scRNA sequencing studies in COVID-19 also provides compelling confirmation of the biomarker expression in immune effector cells, associating specific cellular sources of eNAMPT release during COVID-19 infection and reflect COVID-19 severity and ARDS progression. Measuring plasma eNAMPT levels may serve as a potential strategy for subject stratification in future therapeutic trials, and demonstrated the utility of eNAMPT-neutralizing therapies in future ARDS trials [22].

## Supporting information

S1 Fig Age and circulating biomarkers correlation. (TIF)

S2 Fig No significant geneder differences in biomarkers levels. (TIF)

S3 Fig eNMAPt correlation with ANG-2, IL1RA and IL-6. (TIF)

S1 Table Biomarkers measured by cohort. (DOCX)

S2 Table GSE264196 data set characteristics. (DOCX)
