# Supplementary material for: eNAMPT is a plasma biomarker of COVID-19 severity and ARDS progression linked to increased immune effector cell expression
Source: PLoS One. 2026 Nov 3;21(11):e0360244. doi: 10.1371/journal.pone.0360244 (PMC13730375; doi:10.1371/journal.pone.0360244)
Supplement: Supplementary file 1 [file pone.0360244.s004.docx]

**Table S1. Biomarker Plasma Levels by COVID-19 Cohort**

| **Biomarker** | **U. Arizona** | **U. Miami** | **U. South Florida** | **P value** |
| --- | --- | --- | --- | --- |
| ANG-2 | 116 (5895± 6198) | 61 (**13111**± 27161) | 69 (5081± 4549) | 0.0006 |
| IL-1RA | 116 (**2618**± 3320) | 61 (1772± 2817) | 71 (660.6± 1347) | <0.0001 |
| IL-1β | - | 60 (**0.5281**± 0.7961) | 22 (0.1657± 0.2163) | <0.0001 |
| IL-6 | 116 (23.66± 69.09) | 61 (**64.09**± 245.8) | 66 (23.03± 84.54) | 0.0191 |
| eNAMPT | 116 (**23.02**± 47.26) | 61 (17.99± 36.18) | 71 (12.70± 9.916) | 0.0002 |
| PSGL-1 | - | 61 (**6979**± 6171) | 71 (3438± 2408) | <0.0001 |
| TNF-α | - | 61 (**2.853**±1.777) | 63 (1.797± 1.793) | <0.0001 |
| IL-8 | 116 (29.28± 48.74) | - | - | - |
| MIF | 116 (41274± 20756) | - | - | - |

Number of biomarker readings (n), mean ± standard deviation (SD)
