# Supplementary material for: eNAMPT is a plasma biomarker of COVID-19 severity and ARDS progression linked to increased immune effector cell expression
Source: PLoS One. 2026 Nov 3;21(11):e0360244. doi: 10.1371/journal.pone.0360244 (PMC13730375; doi:10.1371/journal.pone.0360244)
Supplement: Supplementary file 2 [file pone.0360244.s005.docx]

| Table S2. Cryopreserved PBMC from Healthy controls and patients with COVID-19, and IPF. | | |
| --- | --- | --- |
| Group | N | Age |
| **COVID_19** | **3** | **52.0, 2.6** |
| Post-COVID-19-ILD | 5 | 63.8, 11.3 |
| IPF | 6 | 70,6.4 |
| **Controls** | **4** | **42, 2.2** |
