# Supplementary material for: eNAMPT is a plasma biomarker of COVID-19 severity and ARDS progression linked to increased immune effector cell expression
Source: PLoS One. 2026 Nov 3;21(11):e0360244. doi: 10.1371/journal.pone.0360244 (PMC13730375; doi:10.1371/journal.pone.0360244)
Supplement: Peer review history

# Author comment: Author response to Decision Letter 0

**DOI:** 10.1371/journal.pone.0360244.r001

**Transfer Alert**

This paper was transferred from another journal. As a result, its full editorial history (including decision letters, peer reviews and author responses) may not be present.

18 Jun 2026

# Peer review: Decision Letter 0

**DOI:** 10.1371/journal.pone.0360244.r002

**Authors:** You-Yang Zhao

24 Jul 2026

Dear Dr. Casanova,

Please submit your revised manuscript by Sep 07 2026 11:59PM. If you will need more time than this to complete your revisions, please reply to this message or contact the journal office at plosone@plos.org. When you're ready to submit your revision, log on to https://www.editorialmanager.com/pone/ and select the 'Submissions Needing Revision' folder to locate your manuscript file.

- A letter that responds to each point raised by the academic editor and reviewer(s). You should upload this letter as a separate file labeled 'Response to Reviewers'.
- A marked-up copy of your manuscript that highlights changes made to the original version. You should upload this as a separate file labeled 'Revised Manuscript with Track Changes'.
- An unmarked version of your revised paper without tracked changes. You should upload this as a separate file labeled 'Manuscript'.

If you would like to make changes to your financial disclosure, please include your updated statement in your cover letter. Guidelines for resubmitting your figure files are available below the reviewer comments at the end of this letter.

If applicable, we recommend that you deposit your laboratory protocols in protocols.io to enhance the reproducibility of your results. Protocols.io assigns your protocol its own identifier (DOI) so that it can be cited independently in the future. For instructions see: https://journals.plos.org/plosone/s/submission-guidelines#loc-laboratory-protocols. Additionally, PLOS ONE offers an option for publishing peer-reviewed Lab Protocol articles, which describe protocols hosted on protocols.io. Read more information on sharing protocols at https://plos.org/protocols?utm_medium=editorial-email&utm_source=authorletters&utm_campaign=protocols.

As the corresponding author, your ORCID iD is verified in the submission system and will appear in the published article. PLOS supports the use of ORCID, and we encourage all coauthors to register for an ORCID iD and use it as well. Please encourage your coauthors to verify their ORCID iD within the submission system before final acceptance, as unverified ORCID iDs will not appear in the published article. *Only*  the individual author can complete the verification step; PLOS staff *cannot*  verify ORCID iDs on behalf of authors.

We look forward to receiving your revised manuscript.

Kind regards,

You-Yang Zhao

Academic Editor

PLOS One

Journal Requirements:

When submitting your revision, we need you to address these additional requirements.

1. Please ensure that your manuscript meets PLOS ONE’s style requirements, including those for file naming. The PLOS ONE style templates can be found at

https://journals.plos.org/plosone/s/file?id=wjVg/PLOSOne_formatting_sample_main_body.pdf and

https://journals.plos.org/plosone/s/file?id=ba62/PLOSOne_formatting_sample_title_authors_affiliations.pdf

2. We note that the grant information you provided in the ‘Funding Information’ and ‘Financial Disclosure’ sections do not match.

When you resubmit, please ensure that you provide the correct grant numbers for the awards you received for your study in the ‘Funding Information’ section.

3. Thank you for stating the following financial disclosure: [NIH/NHLBI grants P01HL126609 (JGNG), R44HL145930 (JGNG), R01HL141387 (JGNG)].

Please state what role the funders took in the study.  If the funders had no role, please state: ""The funders had no role in study design, data collection and analysis, decision to publish, or preparation of the manuscript.""

If this statement is not correct you must amend it as needed.

Please include this amended Role of Funder statement in your cover letter; we will change the online submission form on your behalf.

4. Thank you for stating the following in the Competing Interests section: [Conflict of Interest Disclosure: Joe GN Garcia is CEO and Founder of Aqualung Therapeutics Corp].

We note that one or more of the authors are employed by a commercial company: [Aqualung Therapeutics Corp.]

1. Please provide an amended Funding Statement declaring this commercial affiliation, as well as a statement regarding the Role of Funders in your study. If the funding organization did not play a role in the study design, data collection and analysis, decision to publish, or preparation of the manuscript and only provided financial support in the form of authors' salaries and/or research materials, please review your statements relating to the author contributions, and ensure you have specifically and accurately indicated the role(s) that these authors had in your study. You can update author roles in the Author Contributions section of the online submission form.

Please also include the following statement within your amended Funding Statement.

“The funder provided support in the form of salaries for authors [insert relevant initials], but did not have any additional role in the study design, data collection and analysis, decision to publish, or preparation of the manuscript. The specific roles of these authors are articulated in the ‘author contributions’ section.”

If your commercial affiliation did play a role in your study, please state and explain this role within your updated Funding Statement.

2. Please also provide an updated Competing Interests Statement declaring this commercial affiliation along with any other relevant declarations relating to employment, consultancy, patents, products in development, or marketed products, etc.

Within your Competing Interests Statement, please confirm that this commercial affiliation does not alter your adherence to all PLOS ONE policies on sharing data and materials by including the following statement: "This does not alter our adherence to PLOS ONE policies on sharing data and materials.” (as detailed online in our guide for authors http://journals.plos.org/plosone/s/competing-interests) . If this adherence statement is not accurate and there are restrictions on sharing of data and/or materials, please state these. Please note that we cannot proceed with consideration of your article until this information has been declared.

Please include both an updated Funding Statement and Competing Interests Statement in your cover letter. We will change the online submission form on your behalf.

5. In the online submission form, you indicated that [Additional data is available from the corresponding author upon reasonable request.].

All PLOS journals now require all data underlying the findings described in their manuscript to be freely available to other researchers, either 1. In a public repository, 2. Within the manuscript itself, or 3. Uploaded as supplementary information.

This policy applies to all data except where public deposition would breach compliance with the protocol approved by your research ethics board. If your data cannot be made publicly available for ethical or legal reasons (e.g., public availability would compromise patient privacy), please explain your reasons on resubmission and your exemption request will be escalated for approval.

6. Your ethics statement should only appear in the Methods section of your manuscript. If your ethics statement is written in any section besides the Methods, please delete it from any other section.

7. We note that you have provided funding information. However, funding information should not appear in the Acknowledgments section or other areas of your manuscript. We will only publish funding information present in the Funding Statement section of the online submission form.

8. Please upload a copy of Supporting Information Figure/Table/etc. Supplementary Table S1, S2, and S3  which you refer to in your text on pages 13,14 and 15.

9. If the reviewer comments include a recommendation to cite specific previously published works, please review and evaluate these publications to determine whether they are relevant and should be cited. There is no requirement to cite these works unless the editor has indicated otherwise.

[Note: HTML markup is below. Please do not edit.]

Reviewers' comments:

Reviewer’s Responses to Questions

**Comments to the Author**

1. Is the manuscript technically sound, and do the data support the conclusions?

Reviewer #1: No

Reviewer #2: Yes

**********

2. Has the statistical analysis been performed appropriately and rigorously?

Reviewer #1: I Don't Know

Reviewer #2: Yes

**********

3. Have the authors made all data underlying the findings in their manuscript fully available?

The PLOS Data policy

Reviewer #1: Yes

Reviewer #2: Yes

**********

4. Is the manuscript presented in an intelligible fashion and written in standard English?

Reviewer #1: Yes

Reviewer #2: Yes

**********

Reviewer #1: This observational human study of eNAMPT and other cytokines in three cohorts of COVID-19 and ARDS is limited by the following concerns:

Major:

-The paper does not appear to add anything new to the literature other than confirmatory findings in a medium size cohort, given that “Elevated eNAMPT has been documented across multiple critical diseases associated with ARDS including, sepsis, trauma, pancreatitis, radiation pneumonitis and COVID-19 infection” and that “The ALT-100 mAb, has recently shown to exhibit excellent safety profiles in both a Phase 1a safety study involving healthy human volunteers and in a Phase 2a clinical trial in patients with ARDS”.

-The statements that, e.g., “this study identifies a panel of biomarkers associated with diagnostic and severity in COVID-19” could be seen as miss-leading and would be more appropriate if the word “identifies” (or equivalent) was replaced with “confirms” (or equivalent).

-Given that “the healthy controls were substantially younger than to all COVID-19 groups”, it is impossible to know whether the increases in eNAMPT or other biomarkers is due to disease onset/progression or age. The demographics in Table 1 should be spilt into controls vs disease.

-The collection of blood at up to 2 days after hospital admission limits the potential use of the biomarkers as prognostic biomarkers.

-The suggestion that eNAMPT is derived from cells with higher expression of NAMPT is somewhat speculative; it would be useful to show if there any data showing positive correlations between NAMPT expression and eNAMPT release from different cell types including PBMCs. Related to this, it is unjustified to conclude “confirmation of the biomarker findings that immune effector cells are the cellular sources of eNAMPT release during COVID-19 infection” when only one cell type (PBMCs) was assessed. In other words, it is an over-statement to suggest that the “cellular sources” of biomarker genes were assessed, given that only one cell type was studied.

Minor:

-It should be clarified throughout the manuscript that the authors did not perform scRNAseq or analyses of lung tissue prospectively but instead analyzed previously-published scRNAseq and lung atlas datasets.

-It is unclear why some biomarkers were not measured in some of the cohorts. Authors should also describe why they think biomarker levels differ significantly across the three cohorts.

-Suggestions that a panel of 9 pre-selected biomarkers is “comprehensive” or equivalent is an over-statement. It should also be stated how the factors in this custom panel were selected.

-In the abstract, there is some vital information missing: “NAMPT expression was increased in circulating CD14+ and CD16+monocytes from COVID-19 subjects [vs what?] and was increased in specific COVID lung tissue cell populations (CD16+ monocytes, cDCs, fibroblasts, endothelium, alveolar macrophages) [vs what?]”

-Figure 4 and throughout the manuscript: What were the controls for the analyses of scRNAseq data on PBMCs from COVID-19 survivors? Control data should be listed first. What about comparisons between survivors versus non-survivors?

-Figure 5 and throughout: What were the controls for the analyses of NAMPT expression on lung tissue from COVID-19 non-survivors? What about comparisons between survivors versus non-survivors?

-Authors could discuss whether and why they think eNAMPT should be used alone or in combination with the many other biomarkers identified by the authors and others, e.g., IL-1β, IL-6, IL-8, TNF-α, IL-1RA, ANG-2).

Reviewer #2: This manuscript evaluates plasma extracellular nicotinamide phosphoribosyltransferase (eNAMPT) as a biomarker of COVID-19 severity and ARDS progression using three independent clinical cohorts and integrates plasma biomarker analyses with publicly available single-cell RNA sequencing (scRNA-seq) datasets. The study is timely, includes a relatively large patient cohort, and benefits from a multicenter design that strengthens the clinical observations. The finding that plasma eNAMPT levels are elevated in patients with severe COVID-19 further supports previous reports implicating eNAMPT in inflammatory lung injury and ARDS.

Overall, the manuscript is well organized and the clinical data are valuable. However, the study is largely descriptive and confirmatory, with limited mechanistic insight beyond previous publications. In addition, the manuscript contains numerous inconsistencies and presentation errors, including discrepancies between figures and figure legends, inaccurate descriptions, and formatting problems. Therefore, substantial revision is required before the manuscript can be considered for publication.

Major Comments

1. Limited novelty

The principal finding that plasma eNAMPT is elevated in patients with severe COVID-19 and ARDS has already been reported by the authors and by other groups. The present study primarily extends these previous observations by including a larger cohort, additional validation cohorts, expanded biomarker analyses, and integration with public scRNA-seq datasets. The authors should clearly define the novel biological insights provided by the current work and distinguish them from their previous publications. Without additional mechanistic evidence, the overall advance appears incremental.

2. Correlation does not establish the cellular source of circulating eNAMPT

The manuscript concludes that circulating immune cells, particularly CD14⁺ monocytes, are likely the major source of plasma eNAMPT because NAMPT transcripts are enriched in monocytes and several lung cell populations in the scRNA-seq datasets. However, transcriptomic data demonstrate gene expression rather than protein secretion. The current data do not demonstrate that CD14⁺ monocytes actively secrete eNAMPT, that circulating monocytes are the dominant source of plasma eNAMPT, or that lung-derived cells significantly contribute to circulating eNAMPT levels. These conclusions should therefore be moderated. Additional experimental validation, such as intracellular protein staining, ELISA of purified cell populations, or ex vivo secretion assays, would substantially strengthen this conclusion.

3. Plasma eNAMPT may represent a general marker of systemic inflammation rather than a COVID-19-specific biomarker

The authors propose eNAMPT as a biomarker of COVID-19 severity. However, elevated plasma eNAMPT has previously been reported in bacterial sepsis, trauma, ARDS, acute pancreatitis, pulmonary fibrosis, radiation-induced lung injury, and other inflammatory disorders. Therefore, increased circulating eNAMPT is unlikely to be specific to COVID-19. The Discussion should more explicitly address this limitation and clarify whether the authors propose eNAMPT as a COVID-19-specific biomarker, an ARDS biomarker, or a more general indicator of inflammatory tissue injury.

4. The scRNA-seq analysis requires further clarification and deeper analysis

The single-cell analysis is interesting but remains relatively superficial. First, the original public dataset contains four experimental groups; however, throughout the manuscript only healthy controls and COVID-19 samples are analyzed, with the exception of Figure 4C. The authors should clearly explain why the remaining groups were excluded and describe the criteria used for data selection and analysis. Second, the number of cells in the COVID-19 group is substantially lower than that in the healthy control group, which may introduce bias into the comparison. The authors should discuss this limitation and, if possible, integrate additional publicly available datasets with more balanced cell numbers to strengthen the analysis. Third, direct comparisons between healthy controls and COVID-19 samples for each cell cluster, for example using split DotPlots or similar visualization methods, would provide a more informative assessment of differential NAMPT expression.

5. The role of endothelial cells deserves further discussion

The scRNA-seq analysis identifies endothelial cells as one of the cell populations expressing NAMPT. Considering the central role of endothelial injury in COVID-19-associated ARDS, the authors should expand the Discussion regarding endothelial activation, vascular barrier dysfunction, endothelial-derived eNAMPT, and the relationship between eNAMPT and established endothelial injury markers such as angiopoietin-2 (ANG-2). If ANG-2 measurements are available, correlation analyses between ANG-2 and plasma eNAMPT would further strengthen the manuscript.

6. Additional adjustment for clinical variables is needed

It remains unclear whether the association between plasma eNAMPT and disease severity remains significant after adjustment for potential confounding variables, including age, sex, body mass index, diabetes, hypertension, corticosteroid treatment, mechanical ventilation, and renal dysfunction. Multivariable logistic regression or similar analyses would considerably strengthen the conclusions.

7. The manuscript requires more careful editing

The manuscript contains several obvious errors that should have been corrected before submission. For example, Figure 4 contains no Panel H, while the label for Panel D is missing. In Figure 5, the figure panels are inconsistent with the corresponding figure legend. These errors reduce the clarity of the manuscript and should be corrected throughout.

8. Several statements are inaccurate or internally inconsistent

Some descriptions are inconsistent with the presented data. For example, the manuscript states that a total of 420 plasma samples were collected from healthy controls (n = 214) and COVID-19-positive patients (n = 277). However, these numbers sum to 491 rather than 420. The authors should carefully verify all reported sample numbers and clearly explain whether certain samples were excluded from specific analyses.

Minor Comments

1. Please clearly define how ARDS was diagnosed in each clinical cohort and indicate whether the Berlin criteria were applied consistently.

2. Receiver operating characteristic (ROC) analyses should include 95% confidence intervals for all reported AUC values.

3. The Discussion should acknowledge that circulating protein concentrations cannot be directly inferred from mRNA expression levels measured by scRNA-seq.

4. In the Introduction, the target antigen recognized by the therapeutic antibody ALT-100 should be clearly introduced.

5. The healthy control group and the COVID-19 cohort differ substantially in age. The authors should discuss the potential impact of age as a confounding factor or adjust for age in the statistical analyses.

6. Figure 3A is never discussed in the Results section and should either be described or removed.

7. The abbreviation "Sy.x" should be defined when it first appears.

8. The manuscript formatting should be standardized throughout. For example, the presentation of confidence intervals should be consistent (e.g., either "CI" or "CI:", but not both).

9. The Figure 1 legend should include the sample size for each protein because different biomarkers appear to have been measured in different numbers of samples.

10. The Figure 2 legend should indicate the sample size for each analysis and define all abbreviations used in the figure.

11. The Figure 3 legend should specify the sample size. In addition, the reported regression coefficient ".0014" should be written as "0.0014."

12. The Figure 4 legend should indicate that the scRNA-seq dataset includes healthy controls in addition to COVID-19 patients.

13. The Figure 5 legend should describe all sample groups included in the analysis rather than only COVID-19 patients. In addition, the current panel labeling appears incorrect. Panel B should be removed, and the current Panels C and D should be relabeled accordingly.

14. All abbreviations used in tables should be defined in the table footnotes.

15. The manuscript would benefit from careful English editing. For example, the sentence, "The present work, we expand these prior findings..." should be revised to "In the present study, we expand these previous findings..."

**********

what does this mean?). If published, this will include your full peer review and any attached files.

If you choose “no”, your identity will remain anonymous but your review may still be made public.

**Do you want your identity to be public for this peer review?**  For information about this choice, including consent withdrawal, please see our Privacy Policy

Reviewer #1: **Yes:**  Colin Evans

Reviewer #2: No

**********

[NOTE: If reviewer comments were submitted as an attachment file, they will be attached to this email and accessible via the submission site. Please log into your account, locate the manuscript record, and check for the action link "View Attachments". If this link does not appear, there are no attachment files.]

To ensure your figures meet our technical requirements, please review our figure guidelines: https://journals.plos.org/plosone/s/figures

You may also use PLOS’s free figure tool, NAAS, to help you prepare publication quality figures: https://journals.plos.org/plosone/s/figures#loc-tools-for-figure-preparation.

NAAS will assess whether your figures meet our technical requirements by comparing each figure against our figure specifications.

# Author comment: Author response to Decision Letter 1

**DOI:** 10.1371/journal.pone.0360244.r003

27 Aug 2026

Journal Requirements

1. Please ensure that your manuscript meets PLOS ONE’s style requirements, including those for file naming.

Answer: The file naming’s has been reviewed and updated.

2. We note that the grant information you provided in the ‘Funding Information’ and ‘Financial Disclosure’ sections do not match.

Answer: The funding information and financial disclosure have been corrected.

3. Thank you for stating the following financial disclosure: [NIH/NHLBI grants P01HL126609 (JGNG), R44HL145930 (JGNG), R01HL141387 (JGNG)].

Please state what role the funders took in the study. If the funders had no role, please state.

Answer: The statement "The funders had no role in study design, data collection and analysis, decision to publish, or preparation of the manuscript." has been added to the funding section and added to the cover letter as indicated.

4. Please provide an amended Funding Statement declaring this commercial affiliation, as well as a statement regarding the Role of Funders in your study.

Answer: A new Funding Statement has been created, clarifying that the funding organization (NIH-NHLBI) did not play a role in the study design, data collection and analysis, decision to publish, or preparation of the manuscript and clarifying that only provided financial support. Dr. Garcia’s commercial association with Aqua Lung Therapeutics has also been disclosed and confirmed that this commercial affiliation does not alter our adherence to all PLOS ONE policies.

5. In the online submission form, you indicated that [Additional data is available from the corresponding author upon reasonable request.].

Answer: Our manuscript utilized data from two data sets that are publicly available (GEO accession number GSE162911, and GSE171668) and detailed in the Methods section. To avoid any misunderstanding we have deleted the sentence that suggested additional data may be kept by the authors.

6. Your ethics statement should only appear in the Methods section of your manuscript. If your ethics statement is written in any section besides the Methods, please delete it from any other section.

Answer: The ethics statement has been included only in the method section.

7. Funding information should not appear in the Acknowledgments section or other areas of your manuscript. We will only publish funding information present in the Funding Statement section of the online submission form.

Answer: As indicated above the funding information has been removed from the acknowledgments section and agree that this appears in the online submission form.

8. Please upload a copy of Supporting Information Figure/Table/etc. Supplementary Table S1, S2, and S3 which you refer to in your text on pages 13,14 and 15.

Answer: Copies of Supporting Information for Tables S1, and Figures S2, S2 and S3 have been uploaded.

9. If the reviewer comments include a recommendation to cite specific previously published works, please review and evaluate these publications to determine whether they are relevant and should be cited. There is no requirement to cite these works unless the editor has indicated otherwise.

Answer: We acknowledge this statement and have evaluated the cited published works, and deem that they remain important to the subject.

REVIEWERS' #1 COMMENTS

10. This observational human study of eNAMPT and other cytokines in three cohorts of COVID-19 and ARDS is limited by the following concerns:

-The paper does not appear to add anything new to the literature other than confirmatory findings in a medium size cohort, given that “Elevated eNAMPT has been documented across multiple critical diseases associated with ARDS including, sepsis, trauma, pancreatitis, radiation pneumonitis and COVID-19 infection” and that “The ALT-100 mAb, has recently shown to exhibit excellent safety profiles in both a Phase 1a safety study involving healthy human volunteers and in a Phase 2a clinical trial in patients with ARDS”.

Answer: We respectfully disagree with the reviewer’s assessment that the present study offers only confirmatory findings without novel scientific contribution. While prior studies have indeed established eNAMPT as a biomarker across several critical illnesses associated with ARDS and have demonstrated the safety profile of ALT-100 in early-phase clinical trials, the current study provides important new insights specifically in COVID-19-associated ARDS, which remains one of the most devastating causes of acute respiratory failure worldwide.

Our study expands upon the existing literature in several significant ways. First, it evaluates a robust and well-characterized cohort of COVID-19 patients across multiple disease severities. Second, and more importantly, we integrate circulating biomarker analyses with transcriptomic interrogation of specific immune cell populations, identifying CD14+ monocytes as a major cellular population associated with elevated expression of NAMPT and related inflammatory mediators. This translational approach linking systemic biomarkers with cell-specific transcriptomic signatures extends beyond simple confirmation of previous observations and provides additional mechanistic insight into the biological pathways underlying severe COVID-19.

11. The statements that, e.g., “this study identifies a panel of biomarkers associated with diagnostic and severity in COVID-19” could be seen as miss-leading and would be more appropriate if the word “identifies” (or equivalent) was replaced with “confirms” (or equivalent).

Answer: We appreciate the reviewers’ suggestion regarding the use of the term “identifies”. To address this concern and avoid overstating novelty, we have revised throughout to replace terms such as "identifies" with more appropriate language including "confirms," "validates," or "demonstrates association," where applicable.

12. Given that “the healthy controls were substantially younger than to all COVID-19 groups”, it is impossible to know whether the increases in eNAMPT or other biomarkers is due to disease onset/progression or age. The demographics in Table 1 should be spilt into controls vs disease.

Answer: We acknowledge the reviewer’s concern that the healthy control group was younger than the COVID-19 cohorts. To evaluate the relationship between age and circulating levels of the inflammatory and vascular biomarkers in COVID-19 subjects, first, we applied eNAMPT vs Age bivariate correlation rho=0.15, n=163, p=0.051 which did not reveal a meaningful correlation. We next applied a multiple variable regression, confirming that overall the model was not statistically significant (F6,24) = 1.13, p = 0.377) and none of the biomarkers were independently associated with age, including eNAMPT (β = 0.157, p = 0.542), IL-1β (β = 2.352, p = 0.562), IL-1RA (β = 0.00046, p = 0.724), IL-6 (β = -0.061, p = 0.298), ANG2 (β = 0.00022, p = 0.553), and TNFα (β = 2.759, p = 0.406). These results indicate that biomarkers elevations evaluated were not explained by age differences, this analysis has been added to the first Results section.

To improve clarity, Table 1 has been expanded to add the controls characteristics as suggested by the reviewer, and supplementary table S1, that contained the demographic characteristics of the controls has been deleted.

13. The collection of blood at up to 2 days after hospital admission limits the potential use of the biomarkers as prognostic biomarkers.

Answer: We respectfully disagree with this interpretation as this timeframe reflects the practical realities of conducting prospective clinical research in critically ill patients and was transparently reported to fully disclose study procedures. Importantly, enrollment and sample collection in hospitalized patients require informed consent from either the patient or a legally authorized representative, making immediate collection at the time of admission or diagnosis frequently impractical. Such delays are common in clinical biomarker studies and do not negate the prognostic value of biomarkers measured early in the disease course. Moreover, our findings demonstrate significant associations between biomarker levels and disease severity despite this unavoidable logistical constraint, supporting the clinical relevance of these measurements.

14. The suggestion that eNAMPT is derived from cells with higher expression of NAMPT is somewhat speculative; it would be useful to show if there any data showing positive correlations between NAMPT expression and eNAMPT release from different cell types including PBMCs. Related to this, it is unjustified to conclude “confirmation of the biomarker findings that immune effector cells are the cellular sources of eNAMPT release during COVID-19 infection” when only one cell type (PBMCs) was assessed. In other words, it is an over-statement to suggest that the “cellular sources” of biomarker genes were assessed, given that only one cell type was studied.

Answer: We appreciate the reviewer’s comments and apologize for the apparent over statement regarding the cellular origin of eNAMPT. The lack of paired circulating biomarker and transcriptomic data from the same patient cohort is an important limitation of this study. Accordingly, we have revised the manuscript to clarify that our analyses do not definitively establish the cellular origin of circulating eNAMPT.

Our objective was to identify cell populations exhibiting elevated expression of NAMPT and related inflammatory genes using independent COVID-19 scRNA-seq datasets. While PBMC datasets were analyzed, these comprise multiple immune cell populations, including monocytes, lymphocytes, NK cells, and dendritic cells. In addition, our analyses included lung-derived datasets, allowing evaluation of the organ most severely affected in ARDS. Therefore, although we cannot conclusively identify all cellular sources of circulating eNAMPT, our findings provide biologically relevant evidence that both systemic immune cells and pulmonary cell populations contribute to the observed inflammatory signature in COVID-19. Thus, while we agree that our findings do not definitively identify all cellular sources of circulating eNAMPT, the combined analysis of systemic immune cells and pulmonary cell populations provides biologically meaningful evidence regarding candidate cellular contributors to the observed biomarker profile during COVID-19 infection. We have revised the text in the discussion accordingly to avoid overstating these conclusions and knowledge this into our limitations.

15. It should be clarified throughout the manuscript that the authors did not perform scRNAseq or analyses of lung tissue prospectively but instead analyzed previously published scRNAseq and lung atlas datasets.

Answer: The paragraph related to the Single Cell RNA sequencing in the methods section specifies the details of the publicly available data sets included in the analysis and states the GEO # for each one of them.

16. It is unclear why some biomarkers were not measured in some of the cohorts. Authors should also describe why they think biomarker levels differ significantly across the three cohorts.

Answer: Multiple factors accounted for the differences the reviewer indicated. This study collected the specimens over time, reason that not all the markers where consistently measures across the cohorts is due to changes on the panel over time, the samples that were processed initially had different set of markers that the later ones, mostly due to optimization related to the updates, i.e PSGL-1 was implemented later, additionally there were sample limitations to run the additional analytes when panel was modified.

17. Suggestions that a panel of 9 pre-selected biomarkers is “comprehensive” or equivalent is an over-statement. It should also be stated how the factors in this custom panel were selected.

Answer: We appreciate the reviewer’s critique and the opportunity to clarify this point. This panel was subjectively selected to incorporate insights of biological processes that are relevant during lung inflammation and ARDS development. IL-6, IL-1β, IL-1RA, eNAMPT, and ANG-2 are key mediators linking inflammation to fibrosis. IL-6 and IL-1β promote NF-κB activation, immune cell recruitment, and fibroblast activation, while eNAMPT amplifies inflammatory signaling as a damage-associated molecular pattern. Elevated IL-1RA reflects a compensatory response to ongoing inflammation, and ANG-2 contributes to endothelial dysfunction and vascular leak. Together, these biomarkers indicate persistent inflammatory and vascular injury pathways that drive fibrotic remodeling and disease progression. This formation has been included at the beginning of the discussion the discussion

18. In the abstract, there is some vital information missing: “NAMPT expression was increased in circulating CD14+ and CD16+monocytes from COVID-19 subjects [vs what?] and was increased in specific COVID lung tissue cell populations (CD16+ monocytes, cDCs, fibroblasts, endothelium, alveolar macrophages) [vs what?]”

Answer: We appreciate the reviewer feedback and the opportunity to improve this relevant section in our manuscript. The Abstract has been corrected clearly indicating the cell types and the tissue origin from their respective comparison groups; healthy controls in PBMCs and Non-COVID-19 in lung tissue.

19. Figure 4 and throughout the manuscript: What were the controls for the analyses of scRNAseq data on PBMCs from COVID-19 survivors? Control data should be listed first. What about comparisons between survivors versus non-survivors?

Answer: Thank you for the opportunity to clarify this. The data sets used for scRNAseq were completely independent. The PBMCs were obtained from COVID-19 survivors and healthy controls, the other had autopsied lung tissue from non survivors that were infected and lung tissue non-infected.

We have revised Figure 4, the Methods, Results and figure legend to identify these groups explicitly.

20. Figure 5 and throughout: What were the controls for the analyses of NAMPT expression on lung tissue from COVID-19 non-survivors? What about comparisons between survivors versus non-survivors?

Answer: Thank you for this constructive critique. We have revised the Results to clarify that the origin is from autopsied COVID Lung Tissues COVID-19 infected compared to control (non-COVID-19). In addition, we have modified the figure 5 panel D, a dot plot stratified by viral status (True or False), replacing the original panel obtained from a separate data set (GEO GSE#171524). To enhance clarity and improve consistency we have added the same plot from the same dataset (GSE162911) that originated Figure 5 panels A-C.

21. Authors could discuss whether and why they think eNAMPT should be used alone or in combination with the many other biomarkers identified by the authors and others, e.g., IL-1β, IL-6, IL-8, TNF-α, IL-1RA, ANG-2).

Answer: We appreciate the opportunity to expand on this relevant observation. This set of plasma biomarker map onto three converging arms of the fibrotic cascade relevant to COVID-19-associated ARDS: (1) cytokine-driven fibroblast/myofibroblast activation and matrix synthesis (IL-1β, IL-6, TNF-α, counterbalanced by IL-1RA), (2) DAMP-driven epithelial/endothelial-to-mesenchymal transition via TLR4/NF-κB and CD74/MAPK signaling (eNAMPT, MIF), and (3) endothelial/vascular destabilization and leukocyte-mediated tissue injury that sustains the remodeling niche (ANG-2, PSGL-1), with IL-8 more specifically reflecting acute neutrophilic injury. The combined use of this panel is justified since each marker demonstrated to have a specific value on disease progression, i.e. eNAMPT, IL-6, and IL-1RA individually reach AUC 0.85–0.96 for diagnosing COVID-19, but discrimination of ARDS versus pneumonia drops to 0.71–0.79 — no single marker fully resolves severity, so combining orthogonal signals (inflammatory, DAMP, vascular) recovers discriminatory power that any one marker loses at the severity-stratification stage. We have included additional text in our discussion, supporting the value to use multiple biomarker panel.

REVIEWER #2:

22. This manuscript evaluates plasma extracellular nicotinamide phosphoribosyltransferase (eNAMPT) as a biomarker of COVID-19 severity and ARDS progression using three independent clinical cohorts and integrates plasma biomarker analyses with publicly available single-cell RNA sequencing (scRNA-seq) datasets. The study is timely, includes a relatively large patient cohort, and benefits from a multicenter design that strengthens the clinical observations. The finding that plasma eNAMPT levels are elevated in patients with severe COVID-19 further supports previous reports implicating eNAMPT in inflammatory lung injury and ARDS.

Overall, the manuscript is well organized and the clinical data are valuable. However, the study is largely descriptive and confirmatory, with limited mechanistic insight beyond previous publications. In addition, the manuscript contains numerous inconsistencies and presentation errors, including discrepancies between figures and figure legends, inaccurate descriptions, and formatting problems. Therefore, substantial revision is required before the manuscript can be considered for publication.

1. Limited novelty

The principal finding that plasma eNAMPT is elevated in patients with severe COVID-19 and ARDS has already been reported by the authors and by other groups. The present study primarily extends these previous observations by including a larger cohort, additional validation cohorts, expanded biomarker analyses, and integration with public scRNA-seq datasets. The authors should clearly define the novel biological insights provided by the current work and distinguish them from their previous publications. Without additional mechanistic evidence, the overall advance appears incremental.

Answer: We thank the reviewer for raising this important point, and as with our response to Critique #10, we respectfully disagree that the present study is primarily confirmatory. Beyond simply extending a known association to a larger cohort, this work provides several distinct novel contributions. The current study provides important new insights specifically in COVID-19-associated ARDS, which remains one of the most devastating causes of acute respiratory failure worldwide. scRNA-seq data showed NAMPT upregulation specifically in fibroblasts, endothelium, and alveolar macrophages in COVID-19 lung tissue, this literature supports framing eNAMPT/TLR4 signaling as a plausible mechanistic bridge between acute-phase biomarker panel and the "failure of regeneration"/persistent remodeling phenotype described in your conclusion — since the same eNAMPT-driven EMT/EndMT pathways documented in radiation- and bleomycin-induced fibrosis models directly implicate these cell populations in matrix deposition.

This work provides several novel contributions:

• First identification of the cellular source of eNAMPT specifically in COVID-19. scRNA-seq PBMCs from survivors showsNAMPT is significantly upregulated in circulating CD14+ monocytes. A finding not previously established for COVID-19.

• First tissue-level mapping of NAMPT expression in human COVID-19 lung. autopsied in lung tissue revealed increased NAMPT expression in specific structural and immune cell populations (neutrophils, CD16+ monocytes, cDCs, fibroblasts, endothelium, alveolar macrophages) relative to healthy lung.

• A biological rationale for therapeutic stratification. Together, these findings support using this biomarker panel — not eNAMPT alone — to identify the specific patient subgroup likely benefiting for eNAMPT-neutralizing therapy.

23. Correlation does not establish the cellular source of circulating eNAMPT

The manuscript concludes that circulating immune cells, particularly CD14⁺ monocytes, are likely the major source of plasma eNAMPT because NAMPT transcripts are enriched in monocytes and several lung cell populations in the scRNA-seq datasets. However, transcriptomic data demonstrate gene expression rather than protein secretion. The current data do not demonstrate that CD14⁺ monocytes actively secrete eNAMPT, that circulating monocytes are the dominant source of plasma eNAMPT, or that lung-derived cells significantly contribute to circulating eNAMPT levels. These conclusions should therefore be moderated. Additional experimental validation, such as intracellular protein staining, ELISA of purified cell populations, or ex vivo secretion assays, would substantially strengthen this conclusion.

Answer: We appreciate the reviewer’s comment and as we stated in response to Critique #14, agree that transcript-level expression does not establish direct association with protein secretion, and that our data do not definitively prove CD14+ monocytes or lung-resident cells are the source of circulating eNAMPT. We have revised the manuscript to moderate this conclusion accordingly.

Our scRNA-seq analyses were designed to identify candidate cellular sources exhibiting elevated NAMPT transcript expression, not to establish secretion or quantify each population’s contribution to circulating eNAMPT. The PBMC dataset encompasses multiple immune populations (monocytes, lymphocytes, NK cells, dendritic cells), among which CD14+ monocytes showed the most pronounced NAMPT upregulation; the separate lung tissue dataset allowed us to additionally examine the organ most directly affected in ARDS. Together, these findings point to circulating monocytes and specific pulmonary cell populations as biologically plausible contributors to the eNAMPT signal, but we agree this is correlative rather than mechanistic evidence, and the revised Discussion now describes these as candidate cellular sources.

We also agree that the absence of paired biomarker and transcriptomic data from the same patients limits our ability to directly link plasma eNAMPT levels to any specific cellular source, and we have added this to the Limitations section.

24. Plasma eNAMPT may represent a general marker of systemic inflammation rather than a COVID-19-specific biomarker

The authors propose eNAMPT as a biomarker of COVID-19 severity. However, elevated plasma eNAMPT has previously been reported in bacterial sepsis, trauma, ARDS, acute pancreatitis, pulmonary fibrosis, radiation-induced lung injury, and other inflammatory disorders. Therefore, increased circulating eNAMPT is unlikely to be specific to COVID-19. The Discussion should more explicitly address this limitation and clarify whether the authors propose eNAMPT as a COVID-19-specific biomarker, an ARDS biomarker, or a more general indicator of inflammatory tissue injury.

Answer: We obviously agree with the reviewer and that eNAMPT is not specific to COVID-19 as we have multiple publications highlighting the utility of eNAMPT as a biomarker in diverse disorders. As referenced in our introduction it has been reported in limited cohorts as associated to clinical conditions (sepsis, trauma, pancreatitis) that are predisposing to ARDS development, consistent with its role as a general DAMP/TLR4 ligand rather than a pathogen-specific marker. We have revised the Discussion to state this explicitly.

To directly answer the reviewer’s question: we propose eNAMPT — and the broader biomarker panel — as an indicator of ARDS severity and progression driven by innate immune/TLR4-mediated inflammatory injury, applicable across the range of conditions that precipitate ARDS, of which COVID-19 is one. The novelty of the present work lies not in claiming COVID-19 specificity, but in being the first study to validate this panel’s severity, and mortality-discriminating performance within a large, multi-site COVID-19 cohort and to link it to cellular expression.

25. The scRNA-seq analysis requires further clarification and deeper analysis

The single-cell analysis is interesting but remains relatively superficial. First, the original public dataset contains four experimental groups; however, throughout the manuscript only healthy controls and COVID-19 samples are analyzed, with the exception of Figure 4C. The authors should clearly explain why the remaining groups were excluded and describe the criteria used for data selection and analysis. Second, the number of cells in the COVID-19 group is substantially lower than that in the healthy control group, which may introduce bias into the comparison. The authors should discuss this limitation and, if possible, integrate additional publicly available datasets with more balanced cell numbers to strengthen the analysis. Third, direct comparisons between healthy controls and COVID-19 samples for each cell cluster, for example using split DotPlots or similar visualization methods, would provide a more informative assessment of differential NAMPT expression.

Answer: We thank the reviewer for this constructive comment. We have substantially revised the scRNA-seq analysis to clarify the rationale for group selection, address unequal cell recovery between groups, and provide direct cell-type-specific comparisons of NAMPT expression.

The public PBMC scRNA-seq dataset contains four clinical groups: healthy controls, acute COVID-19, post-COVID-19 interstitial lung disease, and idiopathic pulmonary fibrosis. Because the primary objective was to determine whether acute COVID-19 is associated with altered circulating NAMPT expression relative to healthy individuals, the principal comparison was restricted to healthy controls and patients with acute COVID-19. The post-COVID-19 ILD and IPF groups represent chronic fibrotic disease states with distinct clinical stages and pathobiology and were therefore not used as comparator groups for the acute COVID-19 analysis. These groups were retained only for descriptive analyses across the broader disease spectrum. This rationale and the criteria used for group selection are now stated explicitly in Methods, Results, and Figure 4 legend.

We agree that the lower number of recovered cells in the acute COVID-19 group could bias pooled cell-level comparisons. Accordingly, the UMAP, cell-composition, violin, and DotPlot panels are now presented as descriptive visualizations only. For formal inference, raw RNA counts were aggregated by donor within each annotated cell population to generate donor-level pseudobulk profiles, and healthy controls were compared with patients with acute COVID-19 using donors as the independent biological replicates. This approach avoids pseudoreplication, retains all available cells, and accounts for differences in sequencing depth and cell recovery through library-size normalization. The revised analysis demonstrated a significant increase in NAMPT expression in CD14⁺ monocytes from patients with acute COVID-19 relative to healthy controls (log₂ fold change = 1.60, FDR = 0.043), whereas the evidence for other less abundant populations was more limited.

To improve direct visualization, Figure 4 was revised to present healthy controls first and acute COVID-19 second and to include split DotPlots with matched cell-type order, gene order, dot-size scale, and expression scale across groups. These panels now allow direct comparison of both average NAMPT expression and the proportion of NAMPT-expressing cells within each corresponding PBMC population. We also added donor-level cell-composition information and cell counts contributing to each analysis and explicitly discuss the small number of acute COVID-19 donors and lower cell recovery as limitations.

We considered validating the findings in an additional public PBMC scRNA-seq cohort. However, we did not directly merge independent datasets because differences in disease severity, sampling time, sample processing, sequencing chemistry, and cell-type annotation could introduce substantial batch effects and confound interpretation. We now acknowledge the absence of independent single-cell validation as a limitation and state that confirmation in a larger, independently processed cohort will be important.

26. The role of endothelial cells deserves further discussion

The scRNA-seq analysis identifies endothelial cells as one of the cell populations expressing NAMPT. Considering the central role of endothelial injury in COVID-19-associated ARDS, the authors should expand the Discussion regarding endothelial activation, vascular barrier dysfunction, endothelial-derived eNAMPT, and the relationship between eNAMPT and established endothelial injury markers such as angiopoietin-2 (ANG-2). If ANG-2 measurements are available, correlation analyses between ANG-2 and plasma eNAMPT would further strengthen the manuscript.

Answer: We thank the reviewer for this valuable suggestion and agree that that the role of endothelial injury in COVID-19 is important to address. Following the reviewer’s recommendation, we expanded the discussion in two ways.

Endothelial activation and endothelial-derived eNAMPT. scRNA-seq analysis of autopsied COVID-19 lung tissue showed increased NAMPT expression specifically within the endothelial cell population, alongside neutrophils, CD16+ monocytes, cDCs, fibroblasts, and alveolar macrophages. Indicating that pulmonary endothelium is not only a target of eNAMPT-driven injury but also a potential contributing source of circulating eNAMPT during severe COVID-19, a dual role consistent with the broader literature on endothelial cells as both effectors and amplifiers of DAMP-driven inflammatory injury.

Relationship between ANG-2 and eNAMPT. eNAMPT- ANG-2 Endothelial/ Tie-axis injury. Using the plasma panel results Spearman correlation between plasma eNAMPT and ANG-2, using the five markers (eNAMPT, ANG-2, IL-6, IL1RA, Il-1b), commonly measured showed eNAMPT is significantly correlated with all four, most strongly with IL-1RA (rho=0.53, n=165, p<0.0001), followed by IL-6 (rho=0.45, n=160, p<0.0001) and IL-1β (rho=0.41, n=33, p=0.018). The correlation with ANG-2 is the weakest, though still statistically significant (rho=0.24, n=164, p=0.0021). when we additionally modeled eNAMPT into a multivariable regression model with IL-6, IL-1β, and IL-1RA, and ANG-2, the association was no longer significant (p=0.883) indicating that ANG-2’s effect disappears once IL-6 is accounted for. These results suggest that eNAMPT and ANG-2 elevation largely co-occur as part of a shared, IL-6-driven systemic inflammatory response rather than reflecting a direct independent biological coupling between eNAMPT and endothelial/vascular, supporting their combined inclusion in the biomarker panel and we have added this analysis and interpretation to a new section to Results and Discussion. We have added PMID: 38345908 to underscore these issues.

27. Additional adjustment for clinical variables is needed

It remains unclear whether the association between plasma eNAMPT and disease severity remains significant after adjustment for potential confounding variables, including age, sex, body mass index, diabetes, hypertension, corticosteroid treatment, mechanical ventilation, and renal dysfunction. Multivariable logistic regression or similar analyses would considerably strengthen the conclusions.

Answer: Thank you for this suggestion. To clarify if the association of eNAMPT-with disease severity remains significant after adjustment, we conducted an analysis adjusting for age, sex, and mechanical ventilation. Our dataset did not include BMI, diabetes, hypertension, corticosteroid treatment, or renal dysfunction. Results were included and a new panel fin Figure 2D was added.

28. The manuscript requires more careful editing

The manuscript contains several obvious errors that should have been corrected before submission. For example, Figure 4 contains no Panel H, while the label for Panel D is missing. In Figure 5, the figure panels are inconsistent with the corresponding figure legend. These errors reduce the clarity of the manuscript and should be corrected throughout.

Answer: We thank the reviewer for identifying these errors. We have carefully reviewed the entire manuscript and corrected the mislabeled figures and legends throughout. In Figure 4, the missing Panel D label was restored and the incorrect reference to Panel H was corrected. Figure 5 figure panels have been reviewed and are now consistent and corresponding to the text in Results.

29. Several statements are inaccurate or internally inconsistent

Some descriptions are inconsistent with the presented data. For example, the manuscript states that a total of 420 plasma samples were collected from healthy controls (n = 214) and COVID-19-positive patients (n = 277). However, these numbers sum to 491 rather than 420. The authors should carefully verify all reported sample numbers and clearly explain whether certain samples were excluded from specific analyses.

Answer: We appreciate the reviewers’ feedback and apologize for this inconsistency. We have corrected this error; the total number of samples were 491.

30. Please clearly define how ARDS was diagnosed in each clinical cohort and indicate whether the Berlin criteria were applied consistently.

Answer: The Berlin Criteria were applied in the diagnosis of ARDS; this has been added in the Methods.

31. The receiver operating characteristic (ROC) analyses should include 95% confidence intervals for all reported AUC values.

Answer: The 95% confidence intervales have been added for all the reported AUC values.

32. The Discussion should acknowledge that circulating protein concentrations cannot be directly inferred from mRNA expression levels measured by scRNA-seq.

Answer: We thank you for this valuable comment. As mentioned in comment #23, transcript-level expression does not establish direct association with protein secretion. To address this concern, we have acknowledged this important point in the Discussion.

33. In the Introduction, the target antigen recognized by the therapeutic antibody ALT-100 should be clearly introduced.

Answer: We appreciate the suggestion. We have added more information related to ALT-100 and introducing it as a humanized monoclonal antibody designed to neutralize eNAMPT.

34. The healthy control group and the COVID-19 cohort differ substantially in age. The authors should discuss the potential impact of age as a confounding factor or adjust for age in the statistical analyses.

Answer: We appreciate the comment. As discussed in comment #12, the potential impact of age as a confounding has been addressed. We have conducted additional analyses to determine whether the increases in eNAMPT or other biomarker was due to disease onset/ progression or age. Details have been included in our response in comment #12 and added to Results.

35. Figure 3A is never discussed in the Results section and should either be described or removed.

Answer: Results have been incorporated in the section related to the figure 3A, which shows the elevated markers identified in non-survivors compared to survivors.

36. The abbreviation "Sy.x" should be defined when it first appears.

Answer: Sy.x denotes the standard error of the estimate, reflecting regression accuracy. Since our results highlight a high value, it is explained in Results in the Figure legend that “variation remains unexplained by this single predictor “. This abbreviation has been defined at first use in Results and the Figure 2 legend.

37. The manuscript formatting should be standardized throughout. For example, the presentation of confidence intervals should be consistent (e.g., either "CI" or "CI:", but not both).

Answer: We have standardized the confidence intervales abbreviation to CI: throughout the manuscript.

38. The Figure 1 legend should include the sample size for each protein because different biomarkers appear to have been measured in different numbers of samples.

Answer: We appreciate the reviewers suggestion. The legend has been modified to include the samples size for each protein. “Total readings analyzed per biomarker for the healthy control cohort and all COVID-19 cohorts respectively were: eNAMPT (n=209 vs. 277), IL-1β (n=111 vs. 82), IL-6 (n=199 vs. 272), IL-8 (n=65 vs. 145), IL-1RA (n=201 vs. 277), MIF (n=45 vs. 145), ANG-2 (n=213 vs. 275), PSGL-1 (n=53 vs. 132), and TNF-α (n=77 vs. 124)”.

39. The Figure 2 legend should indicate the sample size for each analysis and define all abbreviations used in the figure.

Answer: We have incorporated the sample size and defined all the abbreviations into the figure legend. Total readings analyzed per biomarker for the healthy control cohort and all COVID-19 cohorts respectively were: eNAMPT (n=209 vs. 277), IL-1β (n=111 vs. 82), IL-6 (n=199 vs. 272), IL-8 (n=65 vs. 145), IL-1RA (n=201 vs. 277), MIF (n=45 vs. 145), ANG-2 (n=213 vs. 275), PSGL-1 (n=53 vs. 132), and TNF-α (n=77 vs. 124).

40. The Figure 3 legend should specify the sample size. In addition, the reported regression coefficient ".0014" should be written as "0.0014."

Answer: Figure 3 legend now specifies sample size, and the coefficient has been corrected to "0.0014.".

41. The Figure 4 legend should indicate that the scRNA-seq dataset includes healthy controls in addition to COVID-19 patients.

Answer: We appreciate the reviewer’s suggestion, we have corrected Figure 4 legend, indicating that this data set is from peripheral blood mononuclear cell populations from both healthy controls and patients with acute COVID-19.

42. The Figure 5 legend should describe all sample groups included in the analysis rather than only COVID-19 patients. In addition, the current panel labeling appears incorrect. Panel B should be removed, and the current Panels C and D should be relabeled accordingly.

Answer: We appreciate the opportunity to improve the clarity of this figure legend. Figure legend 5 has been edited to specify NAMPT gene expression was obtained from a data set that included single cell RNA sequencing from lung tissue of COVID-19 positives and COVID-19 negatives.

43. All abbreviations used in tables should be defined in the table footnotes.

Answer: All table footnotes have been revised to define every abbreviation used .

44. The manuscript would benefit from careful English editing. For example, the sentence, "The present work, we expand these prior findings..." should be revised to "In the present study, we expand these previous findings..."

Answer: Thank you for the opportunity to improve our manuscript; the full manuscript has been reviewed and edited for grammar and clarity.

Attachment Submitted filename: Response to the Reviewers 08.24.26.docx

# Peer review: Decision Letter 1

**DOI:** 10.1371/journal.pone.0360244.r004

**Authors:** You-Yang Zhao

25 Sep 2026

eNAMPT is a plasma biomarker of COVID-19 severity and ARDS progression linked to increased immune effector cell expression

PONE-D-26-20038R1

Dear Dr. Casanova,

We’re pleased to inform you that your manuscript has been judged scientifically suitable for publication and will be formally accepted for publication once it meets all outstanding technical requirements.

Within one week, you’ll receive an e-mail detailing the required amendments. When these have been addressed, you’ll receive a formal acceptance letter and your manuscript will be scheduled for publication.

An invoice will be generated when your article is formally accepted. Please note, if your institution has a publishing partnership with PLOS and your article meets the relevant criteria, all or part of your publication costs will be covered. Please make sure your user information is up-to-date by logging into Editorial Manager at Editorial Manager® and clicking the ‘Update My Information' link at the top of the page. For questions related to billing, please contact billing support.

If your institution or institutions have a press office, please notify them about your upcoming paper to help maximize its impact. If they’ll be preparing press materials, please inform our press team as soon as possible -- no later than 48 hours after receiving the formal acceptance. Your manuscript will remain under strict press embargo until 2 pm Eastern Time on the date of publication. For more information, please contact onepress@plos.org.

Kind regards,

You-Yang Zhao

Academic Editor

PLOS One

Additional Editor Comments (optional):

Reviewers' comments:

Reviewer’s Responses to Questions

**Comments to the Author**

Reviewer #1: All comments have been addressed

Reviewer #2: All comments have been addressed

**********

2. Is the manuscript technically sound, and do the data support the conclusions?

Reviewer #1: Yes

Reviewer #2: Yes

**********

3. Has the statistical analysis been performed appropriately and rigorously?

Reviewer #1: I Don't Know

Reviewer #2: Yes

**********

4. Have the authors made all data underlying the findings in their manuscript fully available?

The PLOS Data policy

Reviewer #1: Yes

Reviewer #2: Yes

**********

5. Is the manuscript presented in an intelligible fashion and written in standard English?

Reviewer #1: Yes

Reviewer #2: Yes

**********

Reviewer #1: (No Response)

Reviewer #2: The revised manuscript has been substantially improved, and the authors have appropriately addressed the major concerns raised in the previous round review. I just have a minor correction: The label "D" was missed in Fiure4.

**********

what does this mean?). If published, this will include your full peer review and any attached files.

If you choose “no”, your identity will remain anonymous but your review may still be made public.

**Do you want your identity to be public for this peer review?**  For information about this choice, including consent withdrawal, please see our Privacy Policy

Reviewer #1: No

Reviewer #2: No

**********

# Editor report: Acceptance letter

**DOI:** 10.1371/journal.pone.0360244.r005

**Authors:** You-Yang Zhao

PONE-D-26-20038R1

PLOS One

Dear Dr. Casanova,

I'm pleased to inform you that your manuscript has been deemed suitable for publication in PLOS One. Congratulations! Your manuscript is now being handed over to our production team.

At this stage, our production department will prepare your paper for publication. This includes ensuring the following:

* All references, tables, and figures are properly cited

* All relevant supporting information is included in the manuscript submission,

* There are no issues that prevent the paper from being properly typeset

You will receive further instructions from the production team, including instructions on how to review your proof when it is ready. Please keep in mind that we are working through a large volume of accepted articles, so please give us a few days to review your paper and let you know the next and final steps.

Lastly, if your institution or institutions have a press office, please let them know about your upcoming paper now to help maximize its impact. If they'll be preparing press materials, please inform our press team within the next 48 hours. Your manuscript will remain under strict press embargo until 2 pm Eastern Time on the date of publication. For more information, please contact onepress@plos.org.

You will receive an invoice from PLOS for your publication fee after your manuscript has reached the completed accept phase. If you receive an email requesting payment before acceptance or for any other service, this may be a phishing scheme. Learn how to identify phishing emails and protect your accounts at https://explore.plos.org/phishing.

If we can help with anything else, please email us at customercare@plos.org.

Thank you for submitting your work to PLOS One and supporting open access.

Kind regards,

PLOS One Editorial Office Staff

on behalf of

Dr. You-Yang Zhao

Academic Editor

PLOS One
